# Functional significance of gain-of-function *H19* lncRNA in skeletal muscle differentiation and anti-obesity effects

**DOI:** 10.1186/s13073-021-00937-4

**Published:** 2021-08-28

**Authors:** Yajuan Li, Yaohua Zhang, Qingsong Hu, Sergey D. Egranov, Zhen Xing, Zhao Zhang, Ke Liang, Youqiong Ye, Yinghong Pan, Sujash S. Chatterjee, Brandon Mistretta, Tina K. Nguyen, David H. Hawke, Preethi H. Gunaratne, Mien-Chie Hung, Leng Han, Liuqing Yang, Chunru Lin

**Affiliations:** 1 Department of Molecular and Cellular Oncology, The University of Texas MD Anderson Cancer Center, Houston, TX 77030 USA; 2 Current address: Sanofi U.S., Boston, MA 02139 USA; 3 Department of Biochemistry and Molecular Biology, The University of Texas Health Science Center at Houston McGovern Medical School, Houston, TX 77030 USA; 4 Department of Biochemistry and Biology, University of Houston, Houston, TX 77204 USA; 5 Current address: UPMC Genome Center, Pittsburgh, PA 15232 USA; 6 Department of Systems Biology, The University of Texas MD Anderson Cancer Center, Houston, TX 77030 USA; 7 Graduate Institute of Biomedical Sciences, Research Center for Cancer Biology, and Center for Molecular Medicine, China Medical University, Taichung, 404 Taiwan; 8 Department of Biotechnology, Asia University, Taichung, 413 Taiwan; 9 Center for Epigenetics and Disease Prevention, Institute of Biosciences and Technology, Texas A&M University, Houston, TX 77030 USA; 10 Center for RNA Interference and Non-Coding RNAs, The University of Texas MD Anderson Cancer Center, Houston, TX 77030 USA; 11 The Graduate School of Biomedical Sciences, The University of Texas MD Anderson Cancer Center, Houston, TX 77030 USA

**Keywords:** Long noncoding RNA, *H19*, Skeletal muscle, Obesity, RNA therapy, Dystrophin

## Abstract

**Background:**

Exercise training is well established as the most effective way to enhance muscle performance and muscle building. The composition of skeletal muscle fiber type affects systemic energy expenditures, and perturbations in metabolic homeostasis contribute to the onset of obesity and other metabolic dysfunctions. Long noncoding RNAs (lncRNAs) have been demonstrated to play critical roles in diverse cellular processes and diseases, including human cancers; however, the functional importance of lncRNAs in muscle performance, energy balance, and obesity remains elusive. We previously reported that the lncRNA *H19* regulates the poly-ubiquitination and protein stability of dystrophin (DMD) in muscular dystrophy.

**Methods:**

Here, we identified mouse/human *H19-*interacting proteins using mouse/human skeletal muscle tissues and liquid chromatography–mass spectrometry (LC-MS). Human induced pluripotent stem-derived skeletal muscle cells (iPSC-SkMC) from a healthy donor and Becker Muscular Dystrophy (BMD) patients were utilized to study DMD post-translational modifications and associated proteins. We identified a gain-of-function (GOF) mutant of *H19* and characterized the effects on myoblast differentiation and fusion to myotubes using iPSCs. We then conjugated *H19* RNA gain-of-function oligonucleotides (Rgof) with the skeletal muscle enrichment peptide agrin (referred to as AGR-*H19*-Rgof) and evaluated AGR-*H19*-Rgof’s effects on skeletal muscle performance using wild-type (WT) C57BL/6 J mice and its anti-obesity effects using high-fat diet (HFD)- and *leptin* deficiency-induced obese mouse models.

**Results:**

We demonstrated that both human and mouse *H19* associated with DMD and that the *H19* GOF exhibited enhanced interaction with DMD compared to WT *H19*. DMD was found to associate with serine/threonine-protein kinase MRCK alpha (MRCKα) and α-synuclein (SNCA) in iPSC-SkMC derived from BMD patients. Inhibition of MRCKα and SNCA-mediated phosphorylation of DMD antagonized the interaction between *H19* and DMD. These signaling events led to improved skeletal muscle cell differentiation and myotube fusion. The administration of AGR-*H19*-Rgof improved the muscle mass, muscle performance, and base metabolic rate of WT mice. Furthermore, mice treated with AGR-*H19*-Rgof exhibited resistance to HFD- or *leptin* deficiency-induced obesity.

**Conclusions:**

Our study suggested the functional importance of the *H19* GOF mutant in enhancing muscle performance and anti-obesity effects.

**Supplementary Information:**

The online version contains supplementary material available at 10.1186/s13073-021-00937-4.

## Background

Physical exercise is a common strategy for improving muscle performance [1]. Muscle architecture is primarily composed of type I, type IIa, IIb, and IIx fibers, and the arrangement of these fiber types in the musculoskeletal system directly contributes to muscle morphology and physiology [2]. Resistance exercise mostly promotes skeletal muscle hypertrophy and improves strength by increasing the number of type II/glycolytic muscle fibers [3]. The growth and function of skeletal muscle is heavily influenced by hormones. Although the use of steroidal androgens, central nervous system (CNS) stimulants, and metabolic enhancers is frequently considered for improving skeletal muscle growth and performance [4–6], a pharmaceutical approach targeting the stimulation of natural hypertrophic muscular signaling pathways has remained elusive. Dystrophin (DMD) has been shown to be essential in skeletal muscle differentiation and regeneration. The proteomic regulation of DMD remains largely unknown.

Skeletal muscle comprises about 40% of total body weight and accounts for almost 30% of resting energy expenditure [7]. It plays key roles in the regulation of systemic energy homeostasis and metabolic health. Obesity has been implicated in significant alternations to muscle morphology [8, 9] and continues to be a common major health problem in the US, contributing to the development of a multitude of serious health issues, including type-2 diabetes and cancer [10]. Current anti-obesity pharmaceutical approaches primarily target the inhibition of lipogenic processes and appetite or exert influence on metabolic pathways such as those associated with absorption of fatty acids or cellular fatty acid metabolism [11–13]. The aforementioned considerations translate to a diminished ability to exercise under obese conditions, while pharmaceutical approaches fail to address obesity-associated developments in muscle morphology. These insufficiencies have resulted in the urgent need for advances in therapeutic strategies for treating the obesity and restoring a balance between healthy muscular tissue morphologies and patient body mass indices.

Long non-coding RNA (lncRNA) are non-coding RNAs greater than 200 bp with little or no coding potential and are considered the most numerous and functionally diverse class of RNAs [14]. Previous studies have indicated the functional roles of lncRNAs in myogenic differentiation [15]. *H19* is an lncRNA that is highly expressed in adult muscle tissues and is upregulated during myoblast differentiation and regeneration [16, 17]. We previously reported that *H19* is associated with DMD and that impaired *H19*-DMD interactions led to increased E3 ubiquitin-protein ligase TRIM63-mediated, K48-linked poly-ubiquitination of DMD and reduced DMD half-life [18]. Here, we report that a gain-of-function (GOF) mutant of *H19*, referred to as *H19*-GOF, exhibits increased binding affinity toward DMD protein. Serine/threonine-protein kinase MRCK alpha (MRCKα) and α-synuclein (SNCA) were identified as binding proteins associated with DMD in human induced pluripotent stem-derived skeletal muscle cells (iPSC-SkMC) from Becker Muscular Dystrophy (BMD) patients. Disruption of MRCKα and SNCA-mediated phosphorylation of DMD improved *H19*-DMD interactions and further stabilized DMD. Myoblasts expressing the *H19*-GOF mutant showed enhanced differentiation and fusion to myotubes. We developed RNA mimics of *H19-*Rgof conjugated with the muscle-enriching peptide agrin, referred to as AGR-*H19*-Rgof mimics. The administration of AGR-*H19*-Rgof, significantly improved the muscle growth and strength of WT animals with undetectable toxicity. Animals subjected to AGR-*H19*-Rgof treatment were resistant to high-fat diet (HFD)- and *leptin* deficiency-induced obesity. Hence, our data suggested the functional importance of *H19* in skeletal muscle growth and metabolic balance.

## Methods

### In vivo murine models and treatment procedures

The mice used in this study were purchased from Jackson Lab as follows: *Lep*^Ob/Ob^ (stock 000632), C57BL/6 J (stock 000664). All animals were from a C57BL/6 J background and housed with a 12 h light/12 h dark cycle in the animal facility with free access to water and food. 4 to 10 mice were used in each group. Exact numbers for each experiment are included in the figure legends.

To determine the effects of *H19*-Rgof with a skeletal muscle enrichment peptide agrin (AGR-*H19*-Rgof) on WT C57BL/6 J or *Lep*^Ob/Ob^ mice, AGR-tagged oligonucleotides were used. The synthesis and sequence information of AGR-tagged RNA oligonucleotides are described in “Synthesis of *H19*-Rgof mimics, AGR-*H19*-Rgof mimics, and Pharmacokinetics (PK) studies.” AGR-Scr (conjugated to scramble oligonucleotides), *H19*-Rgof, or AGR-*H19*-Rgof mimics were injected at a dose of 1 mg/kg intraperitoneally into 3-week-old male and female mice. For AGR-*H19*-Rgof treatment in HFD (Cat # D12492, Research Diets, Inc.)-fed mice, male C57BL/6 J mice were fed with a HFD starting at 6 weeks of age. The AGR-*H19-*Rgof and AGR-Scr mimics were injected at a dose of 0.5 or 1 mg/kg intraperitoneally every other day starting 2 weeks after the initiation of HFD feeding. Body weight was measured weekly. The general health condition, grooming, and behavior of all animals were monitored daily, and injection sites were checked for signs of redness or edema.

### Synthesis of *H19*-Rgof mimics, AGR-*H19*-Rgof mimics, and pharmacokinetics (PK) studies

The *H19*-Rgof mimics and AGR-*H19*-Rgof mimics were synthesized by Bio-synthesis Inc. The linker that connected the peptide and RNA oligonucleotides was succinimidyl 4-(N-maleimidomethyl) cyclohexane-1-carboxylate (SMCC). RNA mimics sequences are listed in the Additional file 1: Table S1. To characterize the pharmacokinetics of *H19*-Rgof and AGR-*H19*-Rgof mimics, the biotinylated *H19*-Rgof and AGR-*H19*-Rgof mimics (10 mg/kg) were intraperitoneally injected into C57BL/6 J mice. The liver, kidneys, lungs, heart, and skeletal muscles of the animals were collected 3, 12, 24, 48, or 72 h post-injection (*n* = 5 animal per time point). Tissues were subjected to immunohistochemistry using streptavidin alkaline phosphatase conjugates and small RNA isolation in accordance with the manufacturer’s instructions using miRCURY™ RNA Isolation Kits (Qiagen). Immunostained tissue slides were obtained with a Vectra Polaris Multispectral Imaging scanner (PerkinElmer). The quantification of immunohistochemistry staining density was performed by Image-pro plus 6.0 (Media Cybernetics) and calculated based on the average staining intensity and the percentage of positively stained cells. Pharmacokinetic parameters were determined by nonlinear regression analysis.

### Tissue collection

Unless otherwise indicated, mice were fasted for 4–6 h, anesthetized with isoflurane, and sacrificed by heart puncture. Tissues were dissected, weighed, and either dipped in liquid nitrogen or fixed for immunohistochemistry analysis. Individual muscle was dissected on one side of the body and weighed. Adipose tissues including white adipose tissue (WAT), visceral adipose tissue (VAT), and brown adipose tissue (BAT) were collected and weighed.

### Run-to-exhaustion test

Exhaustion treadmill running and sprint running were used to evaluate the physiological activity of the mice. The mice were repeatedly exercised twice weekly for 2 weeks using a treadmill (Ugo Basile, 57631, Stoelting). For the animal exercise, mice were placed on the horizontal treadmill starting at 5 m/min and kept on for about 10 min. The speed was then increased to 10 m/min for the remaining 30 min. Treadmill training was voluntary and followed a standardized protocol [19]. When performing the speed test, the mice were placed on a treadmill with a 15° incline. We increased the speed from 6 m/min to 26 m/min at increments of 2 m/min, which each increment lasting 2 min. Mice were considered to have reached the point of exhaustion when they made contact with the grid for a period of time greater than 5 s. The running speed of the mice was recorded and the animals were returned to their cages once exhausted. For the sprint test: the sprint regimen consisted of mice warming up for 3 min at 6 m/min on a 0° incline, followed by a 2 min run at the same speed with a 15° incline. Subsequent runs were performed at a 15° incline. Speed was first increased to 14 m/min for 30 s, followed by 1.5 min of low speed running at 6 m/min. The speed was then increased to 18 m/min for 30 s. Afterwards, we increased the speed from 18 m/min to 24 m/min at 2 m/min increments, with each increment lasting 15 s (total 15 × 4 = 60 s). After that, the mice continued interval running at 6 m/min for 3 min, followed by an increase in the speed from 20 m/min to 32 m/min at 4 m/min increments, with each increment lasting 15 s. This pattern of exercise continued with subsequent intervals running at 6 m/min for 3 min, followed by a sharp increase in speed from 22 m/min to 34 m/min at 4 min increments, with each increment lasting 15 s. This pattern was repeated with increasing speed until the mice could no longer keep up and gave up on the grid.

### Forelimb grip strength test

A grip strength meter (Columbus Instruments) was used to measure the forelimb grip strength as previously reported [20]. The grid was attached to a force transducer to measure the maximum force applied by the mouse on the grid during the pull. The body weight of the mice was recorded prior to the test and the units of force were calculated in grams-of-force.

### Echocardiography

The heart function of the mice was assessed using functional rodent echocardiography. After their body hair from the neckline to mid chest level was removed with hair removal cream, the mice were anesthetized with 2% isoflurane in the induction chamber and placed in a supine position atop a heating pad to maintain body temperature. The concentration of isoflurane was adjusted to keep a target heart rate of 450 ± 50 beats per minute (bpm). A rectal probe was gently inserted to continuously monitor and adjust body temperature (37.0 °C ± 0.5 °C) via the heating pad. Electrode gel was applied to all four paws, which were then taped to the ECG electrodes. The mice were subjected to transthoracic echo using Vevo 2100 (MS-550D, 22–55 MHz, VisualSonics Inc.).

### Induced pluripotent stem cells (iPSC) maintenance and differentiation and cell cultures

The human skin fibroblasts GM09503 (healthy donor), GM02298 (BMD patient), or GM04569 (BMD patient) were obtained from the Coriell Institute and reprogramed to iPSC by Baylor Human Stem Cell Core. Clinical information is summarized in Additional file 2: Table S2. iPSCs from patients with BMD were used to study the potential molecular mechanisms of DMD stability within pathological context. iPSCs were maintained in feeder-free conditions using the mTeSR™1 medium (Stemcell technologies) on hESC-Qualified Matrigel (Corning) coated plates at 37 °C with 5% CO_2_. *H19* knockout iPSC cell lines were generated using the CRISPR-Cas9 genome editing system, as described previously [18]. iPSCs transfected with sgRNAs were selected with puromycin (250 ng/ml) for 7 days. Emerging iPSC colonies were picked individually and further expanded in mTeSR™1 medium. The presence of targeted gene knockout was confirmed by further genotyping characterization and PCR product sequencing.

For myogenic differentiation, iPSCs were differentiated into skeletal muscle cells using Skeletal Muscle Differentiation Kit (Genea Biocells) according to the manufacturer’s protocol. Briefly, iPSCs were plated onto a collagen I coated plate and maintained for 6–10 days in Skeletal Muscle Induction Medium (Genea Biocells) until confluent. Dissociated myogenic precursors were then plated on a collagen I coated plate and cultured in Skeletal Myoblast Medium (Genea Biocells) for 6–8 days. When confluent, cells were maintained in Myotube Medium or Myotube Fusion Medium (Genea Biocells). Cryopreserved stocks of myoblasts Genea002 were obtained from Genea Biocells. Genea002 was initially maintained in Skeletal Myoblast Medium and changed to Myotube Medium or Myotube Fusion Medium when confluent.

The mouse myoblast cell line C2C12 was purchased from American Type Culture Collection (ATCC) and cultured in DMEM supplemented with 10% FBS. Normal human smooth muscle cells (HSMC) were purchased from Lifeline Cell Technology. The C2C12 *DMD* knockout cell line was generated by transfecting with DMD Double Nickase Plasmid (Santa Cruz), and knockout efficiency was verified by western blot. The C2C12 *H19* knockout cell line was generated using the CRISPR/Cas9 genome editing system through the MD Anderson Cancer Center Gene Editing/Cellular Model Core Facility. sgRNA sequences are listed in the Additional file 1: Table S1. The C2C12 myoblasts were further differentiated to myotubes for the indicated studies.

### Human tissues

All of the human specimen-related studies have been approved by the Institutional Review Board of the University of Texas, MD Anderson Cancer Center. De-identified fresh-frozen human skeletal muscle tissues were commercially obtained from ProteoGenex Inc. All fresh frozen tissue samples are collected under IRB approval by certified medical pathologists. Tissues are snap-frozen post surgery excision. Clinical information is summarized in Additional file 2: Table S2.

### RNA pull-down and liquid chromatography–mass spectrometry (LC-MS) analysis, in vitro RNA-protein binding assay, and dot-blot assay

To identify *H19*-binding proteins, *H19* pulldown was performed as previously described [21]. Briefly, biotin-labeled *H19* RNAs were in vitro transcribed with Biotin RNA Labeling Mix (Roche) and MEGAscript® Transcription Kit (Ambion) then further purified with RNA Clean & Concentrator-5 (Zymo Research). Human skeletal muscle tissues (Additional file 2: Table S2) and mouse skeletal muscle tissue lysates were prepared using the RIPA buffer with anti-RNase, protease/phosphatase inhibitor cocktails supplemented in the lysis buffer. The eluted RNA-protein complexes were denatured, reduced, alkylated, and digested with immobilized trypsin (Promega) for LC-MS analysis at the MD Anderson Cancer Center Proteomics Facility. In vitro RNA-protein binding assay and in vitro RNA pull-down coupled with dot-blot assays were performed as previously described [22]. Briefly, the RNA-capture beads were incubated with recombinant DMD (aa. 3046–3685) protein in binding buffer [50 mM Tris-HCl pH 7.9, 10% Glycerol, 100 mM KCl, 5 mM MgCl_2_, 10 mM β-ME, 0.1% NP-40] for 1 h at 30 °C. Post proteinase K digestion, the RNA fragments were hybridized to the dot-blot with probes reverse complimentary to the human *H19* sequence. Dot-blot probe sequences are listed in the Additional file 1: Table S1.

### Glucose and insulin tolerance tests

Insulin (ITT) and glucose (GTT) tolerance tests were performed on 6-h fasted male and female mice fed with chow or HFD. Glucose values were measured using AimStrip® Plus Blood Glucose Meter (VWR) by tail snip. Glucose (1 g per kg body weight) or human insulin (0.75 U per kg body weight) was injected intraperitoneally (i.p.) after baseline glucose levels were measured in each mouse, and blood glucose levels were measured 15, 30, 45, 60, and 90 min after injection.

### Blood analyses

Whole blood was collected by tail bleeding from un-anesthetized mice or cardiac puncture from mice under deep terminal anesthesia. Mice have been fasted for 4–6 h before blood collection. Total cholesterol (TC) and triglycerides (TG) were determined by enzymatic assays using commercial kits (BioAssay Systems). Alanine aminotransferase (ALT) and aspartate aminotransferase (AST) levels were measured using Alanine Transaminase Colorimetric Activity Assay Kit (700260, Cayman Chemical) and EnzyChrom Aspartate Transaminase Assay Kit (EASTR-100, BioAssay Systems). Serum levels of leptin (ELM-Leptin-1, RayBiotech), IGF1 (RAB0229, Sigma), IGF2 (RAB0231, Sigma), insulin (90080, Crystal Chem), and testosterone (KGE010, R&D) were measured by ELISA assay according to the manufacturer’s instructions.

### Metabolic studies

Whole-body composition parameters were measured in male and female mice fed with either chow or HFD by Faxitron Specimen Radiography System (Faxitron X-Ray Corp.) to precisely measure total body fat and lean mass. Mice were housed in the Oxymax/CLAMS metabolic cage system (Columbus Instruments) for 4 days with ad libitum access to food and water. Volume of oxygen uptake (VO_2_) and exhaled carbon dioxide (VCO_2_), respiratory exchange ratio (RER), and activity were measured using the Oxymax system. RER is determined by dividing VCO_2_ produced by VO_2_ consumed.

### RNAscope, immunohistochemistry image analysis, and quantification

Tissues were fixed in 10% Neutral Buffered Formalin embedded in paraffin, sectioned at 5 μm, and stained with hematoxylin/eosin (H&E). Detection of *H19* expression using a RNAScope probe (designed by Advanced Cell Diagnostics) was performed on mouse tissues with a RNAScope 2.0 High Definition Assay kit according to the manufacturer's instructions (Advanced Cell Diagnostics). The images were visualized with a Zeiss Axioskop2 plus Microscope, and the slides were scanned on the Automated Cellular Image System III for quantification by digital image analysis.

H&E staining was performed in paraffin-embedded livers to visualize the pattern of lipid accumulation and inflammatory status. Lipid droplet accumulation in the liver was visualized using Oil Red O (O0625, Sigma) staining of frozen liver sections prepared in an optimum cutting temperature (O.C.T.) compound (4585, Fisher Scientific). Histopathology images were acquired with a light microscope (Olympus). Adipocyte size (from WAT) was determined with ImageJ software. Sections from five animals per group were used and at least 500 adipocytes per animal were measured from multiple fields.

Skeletal muscles were collected and fixed in 10% Neutral Buffered Formalin at 4 °C for 1 h, followed by 10% sucrose/PBS overnight and then 18% sucrose/PBS overnight at 4 °C before being frozen embedded and sectioned. For immunostaining, frozen cross-sections of muscle were fixed in 4% paraformaldehyde for 5 min, washed, permeabilized with 0.5% Triton X-100, and stained with indicated primary antibodies. After extensive washing in PBS-0.05% Triton X-100, the secondary antibody was added and incubated for 1 h. After extensive washing in PBS-0.05% Triton X-100, nuclei were stained with Vectashield Antifade Mounting Medium with DAPI (Vector Laboratories). Images of laminin-stained fibers were taken by Zeiss microscope. Muscle cross-sectional area and fiber numbers were quantified by ImageJ. Immunostained tissue slides were obtained with Vectra Polaris Multispectral Imaging scanner (PerkinElmer). The quantification of IF staining density was performed by Image-pro plus 6.0 (Media Cybernetics) and calculated based on the average staining intensity and the percentage of positively stained cells. To quantify myotube fusion, fusion index was calculated by dividing the number of nuclei in MHC-positive myotubes with ≥ 3 nuclei by the total number of nuclei analyzed in a field.

Muscle fiber-type immunofluorescence staining was performed with antibodies to MHC type I (supernatant, DSHB, BA-D5), MHC type IIa (supernatant, DSHB, SC-71), and MHC type IIb (supernatant, DSHB, BF-F3), as previously described [23]. Type IIx fibers are not recognized by these antibodies and appeared black. Succinic dehydrogenase (SDH) staining was used to assess metabolic fiber type switch. Frozen cross-sections of muscle were allowed to equilibrate to room temperature and then incubated in a solution containing Nitroblue tetrazolium and sodium succinate for 20 min. Sections were washed 3 times in PBS, dehydrated in ethanol, and mounted with an aqueous mounting medium (Vector Laboratories). The antibodies used are listed in the Additional file 3: Table S3.

### Determination of *K*_*d*_ value using alpha assay

An alpha binding assay was used to determine the *K*_*d*_ for biotinylated *H19*^1951-80^ or *H19*-Rgof and His_6_-DMD or utrophin zinc finger (ZnF) domain, performed as previously described [24]. The *K*_*d*_ was determined by a competition experiment in which unlabeled *H19*^1951-80^ or *H19*-Rgof was titrated (2-fold dilution) as indicated. Streptavidin donor beads and anti-His_6_ AlphaLISA acceptor beads were used in these assays (PerkinElmer). The plate was read on the EnSpire Multimode Plate Reader (PerkinElmer). The competitive inhibition curves were calculated based on alpha signal readings by fitting to a “log (inhibitor) vs. response-variable slope (four parameters)” model (GraphPad Prism 7 software).

### RNA electrophoretic mobility shift assay (EMSA)

The gel mobility shift assay was performed as previously described [25]. Briefly, 500 ng recombinant His-tagged DMD (aa. 3046–3685) protein were incubated with 0.035 pmol ^32^P-labeled WT or mutant probe h*H19*^1951-80^ in a RNA-protein binding buffer (50 mM Tris-HCl 7.9, 10% Glycerol, 100 mM KCl, 5 mM MgCl_2_, 10 mM β-ME, 0.1% NP-40) for 30 min at 30 °C. For cold RNA competition, 0.035 pmol radiolabeled RNA probes were first mixed with 7 pmol cold RNA competitors and then subjected to the gel shift assay, as described above.

### UV-crosslinking and immunoprecipitation (CLIP) and RNA immunoprecipitation (RIP) assay

C2C12 *H19* KO cells were UV crosslinked on ice with three irradiations of 254 nm UV-light at 400 mJ/cm^2^ in a Stratagene crosslinker. CLIP was performed using the DMD antibody as previously described [26]. RNA-protein complexes of interest were then partially purified by immunoprecipitation, and non-covalently associated RNAs were removed by sodium dodecyl sulfate-polyacrylamide gel electrophoresis (SDS-PAGE). RIP assay was performed using Magna RIP™ RNA-Binding Protein Immunoprecipitation Kit (Millipore).

### Mouse L308 array

Serum from AGR-Scr or AGR-*H19*-Rgof-treated mice was collected and analyzed using the mouse L308 antibody array (RayBiotech). Signals for each protein (in duplicate) were obtained by subtracting background signals and normalizing to positive controls. Any ≥ 1.5-fold increase or ≤ 0.65-fold decrease in signal intensity for a single protein between groups was considered a measurable and significant difference in expression.

### Plasmid construction, recombinant protein expression, and transfection

The *H19* sequence was synthesized by GenScript and cloned into the pGEM-3Z vector (Promega) for in vitro transcription and into the pcDNA3.1 (+) vector (Life technologies). An expression vector encoding the wild-type sequence of the human DMD ZnF domain was purchased from the shRNA and ORF core of MD Anderson Cancer Center, and the coding regions were subcloned into the Gateway™ pET-DEST40 vector for mammalian expression and Gateway™ pET-DEST42 vector for prokaryotic expression (Invitrogen). All single-point and deletion mutations were generated using QuikChange™ Lightning Site-Directed Mutagenesis Kit (Agilent Technologies). DMD ZnF Y/F-A mutant substitutes tyrosine (Y) or phenylalanine (F) to alanine (A) at sites Y3326, F3332, Y3334, and F3341. Recombinant DMD WT and mutants were expressed in the *E.coli* strain BL21-CodonPlus (DE3)-RIPL (Agilent Technologies) and purified using a HisPur Cobalt Resin Kit (Thermo Scientific). Plasmid transfections were performed using Lipofectamine3000 (Life Technologies) or electroporation using the 4D-Nucleofector™ System (Lonza) according to the manufacturer’s instructions.

### DNA and RNA isolation, quantitative real-time PCR

Total RNA was isolated from the snap-frozen samples using the RNeasy Plus Mini Kit (Qiagen). cDNA was synthesized from 1 μg of RNA using the iScript™ cDNA Synthesis Kit (Bio-Rad). Quantitative real-time PCR was performed using SsoAdvanced™ Universal SYBR® Green Supermix (Bio-Rad).

The RNA copy number per cell measurement was performed as previously described [27]. Briefly, the total RNA of C2C12 cells with or without *H19* depletion was extracted and detected by qRT-PCR. A standard curve was generated by a serial dilution of in vitro transcribed *H19* RNA, using 728908.2 for human *H19* and 710082.8 for mouse *H19* as the molecular weights, and the total RNA per cell was estimated to be 20 pg.

### Statistical analyses

The experiment was set up to use 3–10 samples/animals/repeats per experiment/group/condition to detect a 2-fold difference with a power of 80% and a significance level of 0.05 using a two-sided test for significant studies. Each of these experiments was independently repeated for 3 times. Results were reported as mean ± standard error of the mean (S.E.M.) or a standard deviation (SD) of at least three independent experiments, as indicated in the figure legends. Each exact *n* value is indicated in the corresponding figure legend. Statistical analysis was performed using GraphPad Prism 7 software. Comparisons were analyzed by unpaired Student’s *t* test, one-way ANOVA test or two-way ANOVA (n.s., *p* > 0.05, *p < 0.05, **p < 0.01, and ***p < 0.001), as indicated in individual figures.

## Results

### *H19*-GOF mutant exhibits enhanced interaction with DMD

**Fig. 1:**
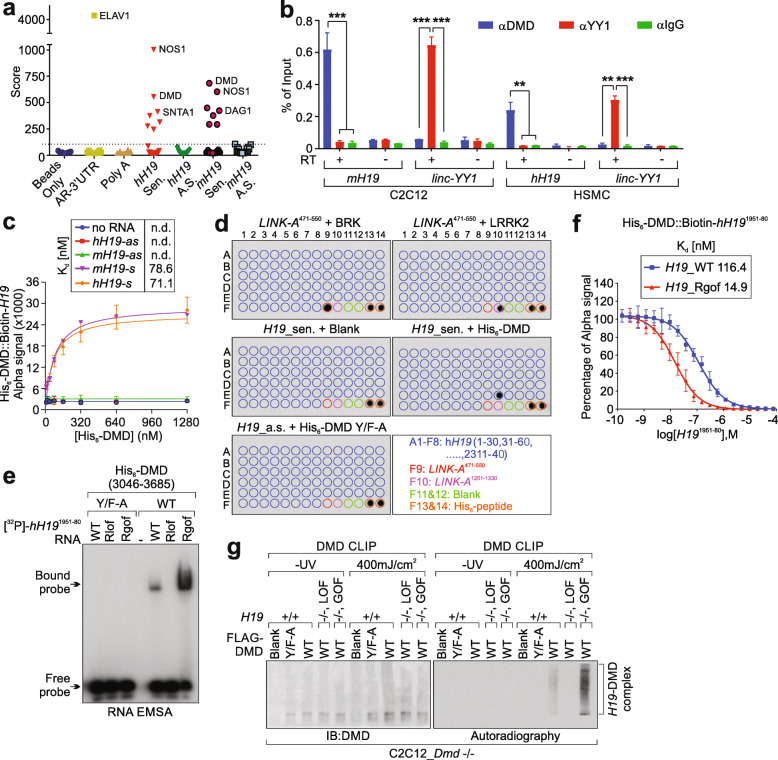
*H19*-GOF mutant exhibits enhanced interaction with DMD. **a** Protein score of human or mouse *H19*-interacting proteins identified by LC-MS. **b** RT-QPCR detection of RIP assay using the indicated antibodies in C2C12 or HSMC. Mean±SEM, *n* = 3 independent experiments, one-way ANOVA. **c** Saturation curve determination using His-tagged recombinant DMD (aa. 3046-3685) and biotinylated *H19*, indicated as a donor/acceptor pair. The concentration of His-tagged recombinant DMD (aa. 3046–3685) is shown. Mean ± SD, *n* = 3 independent experiments. **d** In vitro RNA-protein binding followed by dot blot assays using in vitro transcribed biotinylated *H19* sense (sen.) or antisense (a.s.) in the presence of indicated recombinant proteins. *LINK-A* was included as negative control. BRK (breast tumor kinase) is a confirmed binding partner of *LINK-A*. Bottom panel: annotation for each dot. A1-F8 indicates individual *H19* probes (antisense oligos, 30 bp per probe, from 1 to 2340), F9 indicates antisense sequences of *LINK-A* 471–550, F10 indicates antisense sequences of *LINK-A* 1251–1330, F11 and F12 are blank, and F13 and F14 are His_6_-peptide. **e** EMSA using His-tagged DMD (aa.3046–3685) or Y/F-A mutant and [γ-^32^P]-labeled human *H19* RNA (nt. 1951–1980), Rlof, or Rgof as indicated. **f** Competition binding assay to determine the K_d_ of the interaction between His-tagged DMD (aa.3046–3685) and biotinylated-*H19*^1951-80^. Unlabeled *H19*^1951-80^ WT or Rgof mutant served as competitors. Mean±SD, *n* = 3 independent experiments. **g** Autoradiography (right) or IB detection using the indicated antibodies (left) of CLIP assay in *Dmd-* or *H19*-knockout C2C12 cells expressing the indicated constructs. No significance [n.s.], *p* > 0.05, *, *p* < 0.05, **, *p* < 0.01, ***, *p* < 0.001

RNAscope indicated that h*H19* is mainly expressed in adult skeletal muscle and cardiac muscles but was undetectable in the other mouse organs we tested (Additional file 4: Fig. S1a). To understand the functional role of *H19* in skeletal muscle, we attempted to identify the binding proteins of m*H19/*h*H19* using biotinylated mouse/human *H19* transcripts and mouse/human skeletal muscle tissues, respectively (Fig. 1a and Additional file 2: Table S2). Open-ended LC-MS analysis indicated that both mouse and human *H19* sense transcripts, but not anti-sense transcripts, associated with DMD complex components, including DMD, NOS1 (Nitric oxide synthase), SNTA1 (syntrophin alpha 1), DAG1 (beta-dystroglycan), SGCA (alpha-sarcoglycan), and SNTB1/2 (Beta-1-syntrophin/ Beta-2-syntrophin) [28] (Fig. 1a and Additional file 5: Table S4). The interactions between *H19*-sense, DMD, and DMD complex components in human skeletal muscle, HSMC, and mouse myoblast cells (C2C12) were confirmed (Fig. 1b and Additional file 4: Fig. S1b).

We previously indicated that *H19* associates with the ZnF domain of DMD at the C-terminus (aa. 3046–3685) [18]. Recombinant DMD C-termini exhibit robust interactions with in vitro transcribed human or mouse *H19*-sense RNA, with Kd values of 71.1 and 78.6 nM, respectively (Fig. 1c). To demonstrate the RNA motifs involved in the interactions with DMD, we conducted dot-blot assays [29], which suggested that the human *H19* nt. 1951–1980 associates with DMD (Fig. 1d). AT-rich motifs play essential roles in mediating interactions between RNAs and ZnF domains [30]. The human *H19* RNA motif (nt. 1951–1980) contains two putative AT-rich motifs: nt. 1954-1957 (TGTT) and nt. 1970-1973 (TGTC). We reasoned that mutations of G1955A and G1971A (referred to as GOF mutants) might further increase the presence of AT rich motifs in this RNA region, in contrast to a LOF mutant [18]. EMSA assay using γ-^32^P-labeled RNA oligonucleotides suggested that RNA oligonucleotides representing WT *H19*^1951-1980^ exhibit associations with recombinant DMD C-termini (Fig. 1e). Furthermore, *H19*-GOF oligonucleotides exhibited augmented associations with DMD C-termini (Fig. 1e). Alpha assay quantitatively determined the binding affinities between WT *H19* or the Rgof mutant in interacting with DMD C-termini, with *Kd* values of 116.4 nM and 14.9 nM, respectively (Fig. 1f).

To demonstrate the specificity between the DMD ZnF domain and *H19*, we applied mouse C2C12 myoblast cells with a depleted *DMD* gene and reintroduced a Flag-tagged DMD ZnF domain (referred to as WT) (Fig. 1g and Additional file 4: Fig. S1c). Under *DMD*-deficiency conditions, the *H19* gene was further depleted, and WT *H19*, LOF, or GOF mutants were reintroduced as a rescue experiment. Rescue CLIP assays indicated that WT *H19* exhibits robust interactions with exogenous DMD (Fig. 1g, right panel, lane 8). Upon depletion of *DMD* or expression of DMD Y/F-A mutant, the RNA:DMD complex revealed by CLIP autoradiography was abolished (Fig. 1g, right panel, lane 6, 7). Furthermore, the *H19*-GOF mutants, but not the LOF mutant, exhibited increased association with exogenous DMD (Fig. 1g, right panel, lane 9, 10).

### MRCKα and SNCA facilitate the phosphorylation and poly-ubiquitination of DMD *1*

**Fig. 2:**
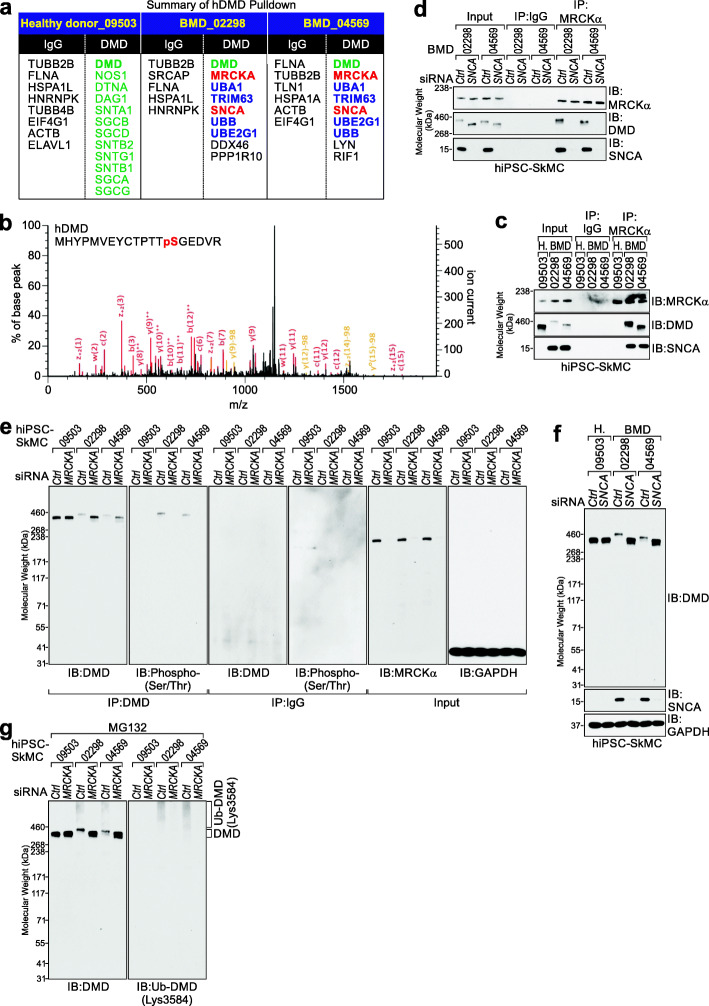
MRCKα and SNCA facilitate the phosphorylation and poly-ubiquitination of DMD. **a** List of DMD-associated proteins identified by LC-MS in iPSC-SkMC derived from GM09503 (healthy donor), GM02298 (BMD patient), or GM04569 (BMD patient), respectively. **b** LC-MS annotation of peptide harboring p-DMD Ser3365. **c** Co-IP followed by immunoblotting (IB) using the indicated antibodies in iPSC-SkMC. **d** Co-IP and IB detection using the indicated antibodies in iPSC-SkMC in the presence of the indicated siRNAs. **e**, **f** IB detection using the indicated antibodies in iPSC-SkMC in the presence of the indicated siRNAs. **g** IB detection using the indicated antibodies in iPSC-SkMC in the presence of the indicated siRNAs and MG132

We previously demonstrated that *H19* modulates ubiquitinated DMD (ub-DMD, K3584) and the protein stability of DMD [18]. To further demonstrate the molecular mechanism of *H19* in modulating post-translational modifications (PTM) other than Ub-DMD, we reasoned that BMD would serve as a suitable candidate given the notion that BMD patients generally harbor reduced, yet detectable DMD protein levels [18]. We immunoprecipitated endogenous DMD proteins from iPSC-SkMC derived from GM09503 (healthy donor), GM02298 (BMD patient), or GM04569 (BMD patient) (Additional file 6: Table S5). DMD and DMD-associated proteins were subjected to LC-MS (Fig. 2a). We focused on the phosphorylation of Serine (S), Threonine (T), and Tyrosine (Y) residues and GG (double glycine) modifications of Lysine (K), with GG most notably suggesting ubiquitin modification potential [31]. We first validated the identification of DMD in GM09503_DMD, GM02298_DMD, and GM04569_DMD samples (Fig. 2a). Interestingly, in GM02298_DMD and GM04569_DMD samples, MRCKα and SNCA were detected as the most confident protein targets (Fig. 2a), suggesting that DMD protein might be subjected to serine/threonine phosphorylation. Figure 2b shows the phosphorylation of DMD Ser3365 detected in GM_02298 and GM_04569 by LC-MS (Additional file 6: Table S5).

To characterize the potential MRCKα-mediated phosphorylation of DMD, we first confirmed that MRCKα, DMD, and SNCA form a protein complex in BMD iPSC-SkMC (Fig. 2c). Co-immunoprecipitation (co-IP) assay indicated that the interactions between MRCKα and DMD are mediated by SNCA (Fig. 2c, d). We noticed that DMD in BMD iPSC-SkMC exhibited elevated molecular weight. We reasoned that phosphorylated DMD might exhibit an apparent molecular weight shift in SDS-PAGE gel, possibly due to reduced SDS binding [32, 33]. The presence of p-DMD Ser3365 in BMD iPSC-SkMC suggested that p-DMD Ser3365 may serve as one of the important modifications in regulating DMD stabilization. To verify the hypothesis that p-DMD Ser3365 is mediated by MRCKα, we found that depletion of *MRCKA* led to diminished phosphorylation of DMD in BMD iPSC-SkMC (Fig. 2e, second panel) with the concurrent restoration of DMD protein levels (Fig. 2e, first panel). Similarly, removal of *SNCA* resulted in enhanced DMD protein status and reduced Ub-DMD, as revealed by a modification specific antibody, in the BMD iPSC-SkMC cells we tested (Fig. 2f, g). Taken together, our findings suggested that SNCA mediates the association between MRCKα and DMD, leading to p-DMD Ser3365 and DMD instability.

SNCA plays important roles in Parkinson’s disease through mediating the aggregation of protofibrils [34]. Although relatively low mRNA levels of SNCA are detected in skeletal and cardiac muscles, our results suggested that the expression levels of SNCA might be increased in BMD patients due to an unknown mechanism, thereby contributing to muscular dystrophy. SynuClean-D (SC-D) is an inhibitor of SNCA aggregation that has been showed to disrupt amyloid fibrils and prevent neuron degeneration [35]. Interestingly, SC-D treatment reduces SNCA aggregation in muscle tissues [35]. MRCKα is a serine/threonine kinase related to the myotonic dystrophy protein kinase (DM-PK) [36], which regulates cytoskeletal reorganization [37]. One small molecule inhibitor, BDP5290 (BDP), has been shown to act as a potent inhibitor targeting MRCKα, with an IC_50_ of 10 nM [38]. We overexpressed SNCA in iPSC-SkMC derived from a healthy donor or BMD patients, finding that expression of SNCA led to phosphorylation of DMD, which was attenuated by SC-D (Additional file 4: Fig. S2a-b). Treatment with SC-D or BDP significantly improved the interaction between *H19* and DMD, but showed minimal effects on the interaction between HuR and Actin [39] (Additional file 4: Fig. S2c-e). Moreover, treatment with SC-D or BDP improved DMD protein levels and reduced DMD phosphorylation and Ub-DMD in BMD iPSC-SkMC (Additional file 4: Fig. S2f-g).

### *H19*-GOF enhances skeletal muscle cell differentiation and fusion

**Fig. 3:**
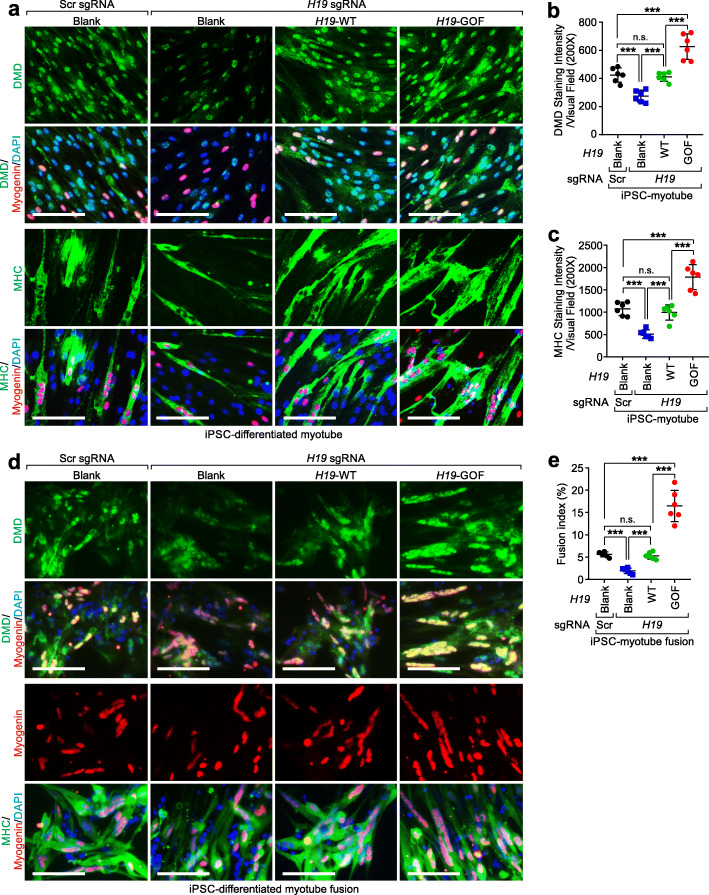
*H19*-GOF mutant promotes myotube differentiation and fusion. **a** Immunofluorescence staining using the indicated antibodies of iPSC-differentiated, *H19*-proficient or *H19*-deficient myotubes, with expression of backbone plasmid (Blank), *H19* WT, or *H19*-GOF plasmid. Scale bars, 100 μm. **b**, **c** Statistical analysis of DMD (b) or MHC (c) staining intensities of iPSC-differentiated, *H19*-proficient or *H19*-deficient myotubes, with expression of indicated plasmid. Mean±SD, *n* = 6 independent experiments, one-way ANOVA. **d** Immunofluorescence staining using the indicated antibodies of iPSC-differentiated, *H19*-proficient or -deficient myotube fusion, with expression of indicated plasmid. Scale bars, 100 μm. (**e**) Percentage of fusion index of iPSC-differentiated, *H19*-proficient or *H19*-deficient myotube fusion, with expression of indicated plasmid. Mean ± SD, *n* = 6 independent experiments, one-way ANOVA. No significance [n.s.], *p* > 0.05, *, *p* < 0.05, **, *p* < 0.01, ***, *p* < 0.001

To determine the effects of the *H19*-GOF mutant, we applied iPSCs from a healthy donor (Additional file 2: Table S2) upon *H19* depletion, followed by the reintroduction of WT or GOF *H19* to iPSCs. These iPSCs were differentiated to myoblasts, which were further differentiated to myotubes (Fig. 3a and Additional file 4: Fig. S3a). Myotubes harboring *H19* sgRNAs exhibited reduced expression of DMD and myosin heavy chain isoform (MHC) (Fig. 3a). Expression of WT *H19* alleviated the DMD and MHC deficiency (Fig. 3a–c). Furthermore, myotubes expressing *H19*-GOF showed increased expression status of DMD and MHC (Fig. 3a–c). We then determined the fusion efficacy of myotubes expressing WT *H19* or the GOF mutant, finding that *H19*-deficiency led to impaired myotube fusion. On the contrary, expression of *H19*-GOF enhanced the myotube fusion percentage (Fig. 3d, e). The expression of WT *H19* or the GOF mutant had minimal effects on the mRNA status of *DMD* in iPSC-differentiated myotubes (Additional file 4: Fig. S3b).

**Fig. 4:**
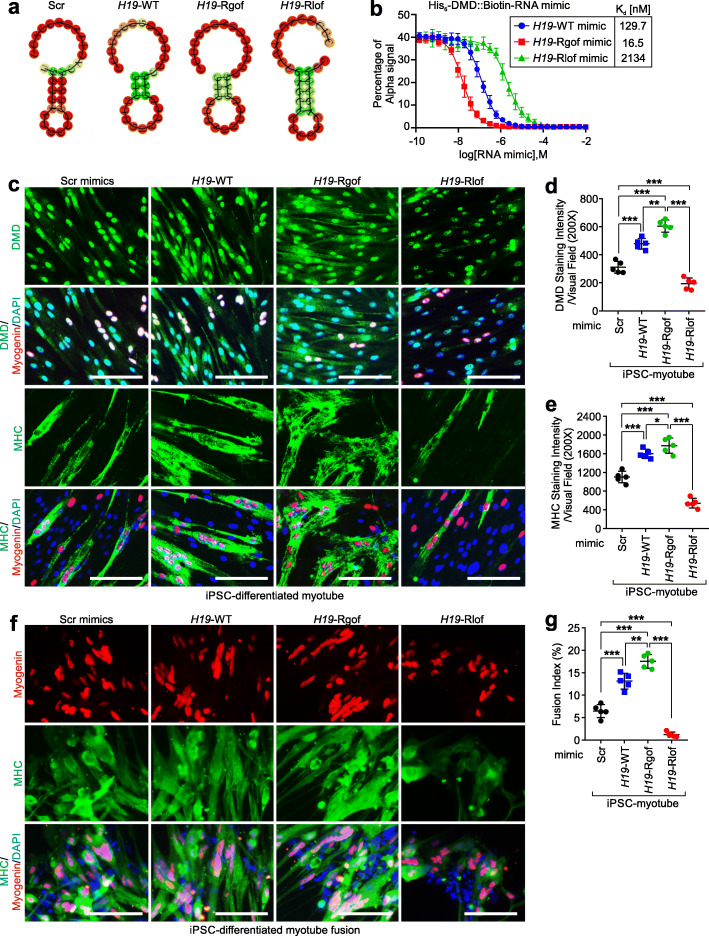
*H19*-Rgof mimics facilitate myotube differentiation and fusion. **a** Graphic illustration of the indicated RNA mimics predicted by RNAfold WebServer. **b** Competition binding assay to determine the K_d_ of the interaction between DMD (aa.3046–3685) and *H19* WT, Rgof, or Rlof mimics. Unlabeled indicated RNA mimics served as competitors. Mean ± SD, *n* = 3 independent experiments. **c** Immunofluorescence staining using the indicated antibodies of iPSC-differentiated myotubes treated with Scr, *H19*-WT, *H19*-Rgof, or *H19*-Rlof mimics. Scale bars, 100 μm. **d**, **e** Statistical analysis of DMD (**d**) or MHC (**e**) staining intensities of iPSC-differentiated myotubes with indicated treatment. Mean±SD, *n* = 5 independent experiments, one-way ANOVA. **f** Immunofluorescence staining using the indicated antibodies of iPSC-differentiated myotubes with indicated treatment. Scale bars, 100 μm. **g** Percentage of fusion index of iPSC-differentiated myotubes with indicated treatment. Mean ± SD, *n* = 5 independent experiments, one-way ANOVA. No significance [n.s.], *p* > 0.05, *, *p* < 0.05, **, *p* < 0.01, ***, *p* < 0.001

We initially determined the minimal *H19* RNA motifs required for interactions with DMD and demonstrated that the *H19*-GOF mutant exhibits enhanced interaction with DMD using dot-blot assays. We therefore aimed to determine the effects of RNA oligonucleotides using RNA mimics representing *H19* WT, GOF, or LOF mutants. The secondary structures of *H19* WT, Rgof, or Rlof mutants are similar (Fig. 4a). We determined the binding affinities of RNA mimics representing *H19* WT, Rgof mimics, or Rlof mimics, respectively, finding that *H19*-WT mimics exhibit adequate binding affinities toward recombinant DMD-CT, with a Kd value of 129.7 nM (Fig. 4b). *H19*-Rgof mimics showed increased binding affinity toward DMD, with a Kd value of 16.5 nM, while *H19*-Rlof mimics exhibited impaired interactions with DMD (Fig. 4b).

We reasoned that *H19*-Rgof mimics may facilitate the protein stabilization of DMD protein. We first applied FITC-conjugated RNA mimics, including Scr, *H19*-WT, *H19*-Rgof, or *H19*-Rlof mimics to the iPSC-differentiated myoblasts, finding that the FITC-conjugated RNA mimics were adequately delivered to the iPSC-differentiated myoblasts (Additional file 4: Fig. S3c). Upon treatment with Scr, *H19*-WT, *H19*-Rgof, or *H19*-Rlof mimics, the myotubes exhibited similar expression statuses of *H19* or DMD, respectively (Additional file 4: Fig. S3d-e). iPSC-differentiated myoblasts were treated with Scr, WT, Rgof mimics, or Rlof mimics, which were further differentiated into myotubes (Fig. 4c). The presence of *H19*-Rgof mimics significantly improved the expression status of DMD and MHC. On the contrary, the myotubes exhibited reduced expression of DMD and MHC following the *H19*-Rlof mutant treatment (Fig. 4c–e). Furthermore, the *H19*-Rgof mutant treatment promoted the fusion index of iPSC-differentiated myotubes (Fig. 4f, g).

### *H19*-Rgof lncRNA mimics improve performance

**Fig. 5:**
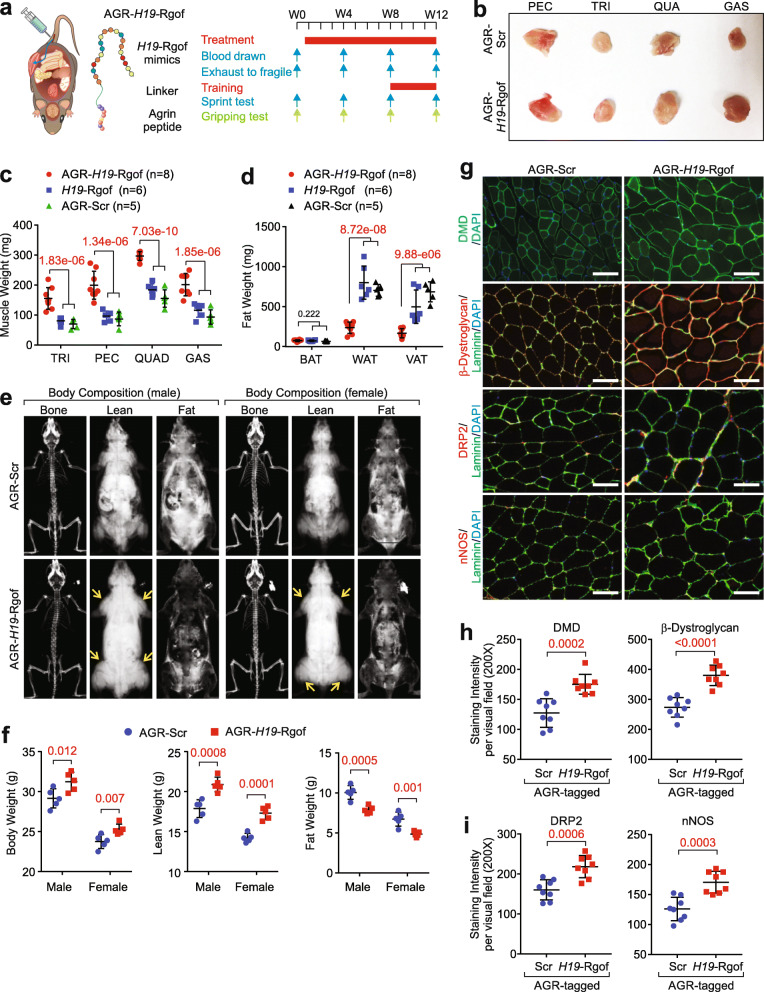
AGR-*H19*-Rgof enhances animal muscle mass. **a** Graphic illustration of AGR-*H19*-Rgof and experimental settings. **b** Representative pictures of AGR-Scr or AGR-*H19*-Rgof-treated muscle pieces. **c** Weights of individual indicated muscles from mice with indicated treatment. Mean±SD, *n* = 5, 6, 8 animals, one-way ANOVA. **d** Weights of BAT, WAT, or VAT from mice with indicated treatment. Mean±SD, *n* = 5, 6, 8 animals, one-way ANOVA. **e** Representative images of lean, fat, and bone tissues by the dual-energy x-ray absorptiometry imaging system from mice with indicated treatment. **f** Quantification of body weight, lean weight, and fat weight by the dual-energy x-ray absorptiometry imaging system from mice with indicated treatment. Mean ± SD, *n* = 5 animals per experimental group, Student’s *t* test. **g** Immunofluorescence staining using the indicated antibodies of TA. Scale bars, 100 μm. **h**, **i** Statistical analysis of staining intensities of DMD (**h**, left), β-dystroglycan (**h**, right) or DRP2 (**i**, left), or nNOS (**i**, right) of TA. Mean ± SD, *n* = 8 animals per experimental group, Student’s *t* test

We reasoned that RNA oligonucleotides representing the *H19*-GOF mutant might improve the animals’ skeletal muscle differentiation, muscle strength, performance, and obesity resistance in vivo. Inspired by peptide-facilitated macromolecular delivery [40], we applied an agrin-derived peptide to improve the in vivo muscle-enriched distribution of *H19*-Rgof mimics [41]. Previous studies identified that Acetylcholine receptor (AChR) clustering is orchestrated by a complex containing agrin, LRP4 (low-density lipoprotein receptor-related protein 4), and MuSK (muscle-specific kinase) [42–44]. Particularly, an eight amino acid insert of agrin (ELTNEIPA, referred to as Z8 insert) plays critical roles in interacting with LRP4 and AChR-clustering [43, 45]. Hence, peptides representing agrin (8 amino acid peptide × 2) were synthesized and linked to *H19*-Rgof mimics, referred to as AGR-*H19*-Rgof (Fig. 5a). We then determined the concentration of biotin-labeled AGR-*H19*-Rgof mimics in the liver, kidneys, lungs, skeletal muscle, and heart post-i.p. administration, finding that a substantial proportion of AGR-*H19*-Rgof mimics were detected in skeletal muscle (Additional file 4: Fig. S3f-g). The liver, kidneys, and lungs exhibited minimal accumulation of biotin-labeled AGR-*H19*-Rgof (Additional file 4: Fig. S3f-g). The skeletal muscle distribution of AGR-*H19*-Rgof was significantly enhanced compared to unlabeled *H19*-Rgof (Additional file 4: Fig. S3f-g).

Utrophin and DMD are structurally and functionally similar [46]. We hence determined the binding affinity between the ZnF domain of DMD or utrophin and *H19*-Rgof. Our findings suggested that the DMD ZnF domain exhibits a binding affinity of 31.4 nM (Additional file 4: Fig. S4a). The DMD C3340Y mutant exhibited an impaired binding affinity with *H19*-Rgof (Additional file 4: Fig. S4a), which was consistent with our findings. Interestingly, the utrophin ZnF domain exhibited adequate interactions with *H19*-Rgof, with a Kd value of 128.3 nM (Additional file 4: Fig. S4a). We reasoned that the administration of AGR-*H19*-Rgof may also facilitate the stabilization of utrophin in skeletal muscles. iPSC-SkMC exhibited elevated utrophin protein status in the presence of AGR-*H19*-Rgof (Additional file 4: Fig. S4b-c).

We then administered AGR-Scr, *H19*-Rgof, or AGR-*H19*-Rgof mimics to 3-week-old male or female mice (1 mg/kg, i.p., every 3 days) for 12 weeks. Compared to AGR-Scr or *H19*-Rgof, animals subjected to AGR-*H19*-Rgof mimic administration exhibited increased muscle weight and reduced fat weight (Fig. 5b–d). Body composition analysis indicated that animals subjected to AGR-*H19*-Rgof mimics exhibited increased body weight, lean weight, and reduced fat weight and fat percentage compared to animals administered AGR-Scr mimics (Fig. 5e, f). The tibialis anterior (TA) of animals supplied with AGR-*H19*-Rgof mimics exhibited increased DMD protein status and improved protein status of the DMD-associated protein complex components β-dystroglycan, DRP2, and nNOS (Fig. 5g–i).

Treatment with AGR-Scr, *H19*-Rgof, or the AGR-*H19*-Rlof mutant showed minimal effects on the expression of *H19* or the *Dmd* transcript in TA (Additional file 4: Fig. S5a-b). Major organs and complete blood cell count (CBC) analysis of animals subjected to RNA mimics indicated minimal signs of toxicity (Additional file 4: Fig. S5c and Additional file 7: Table S6). Mouse serum cytokine array indicated that animals subjected to AGR-Scr or AGR-*H19*-Rgof mimics exhibited similar statuses of IGF1, IGF2, leptin, and testosterone (Additional file 4: Fig. S5d-h). Furthermore, animals treated with AGR-*H19*-Rgof harbored increased utrophin protein status in the quadriceps (QUAD) compared to the animals injected with AGR-Scr RNA oligonucleotides (Additional file 4: Fig. S5i-j).

**Fig. 6:**
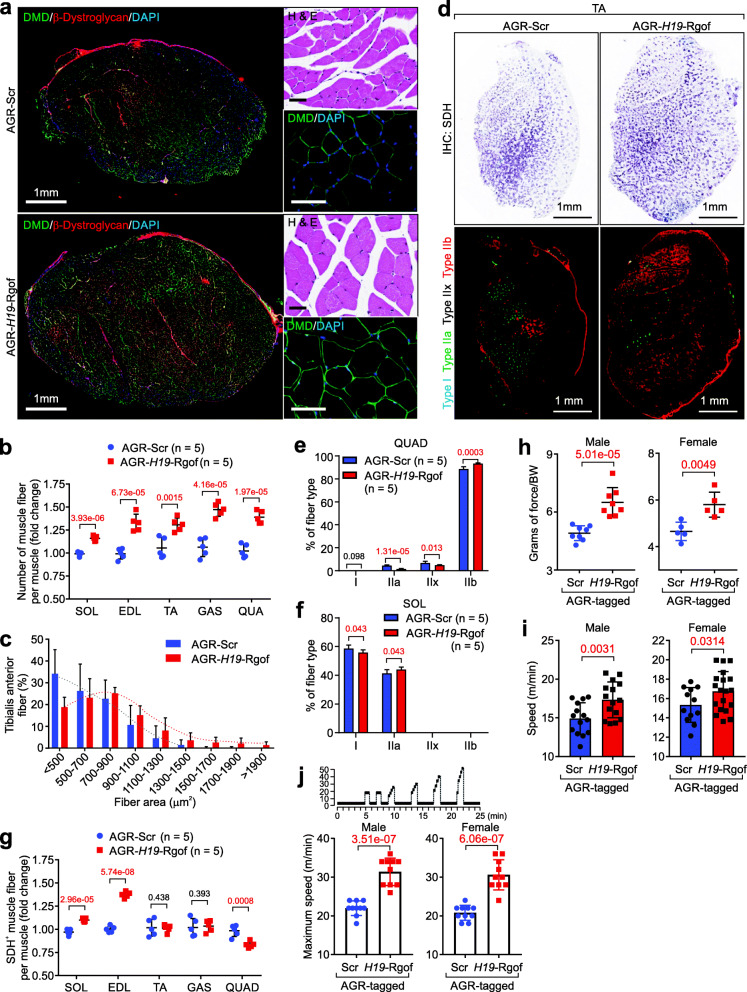
AGR-*H19*-Rgof alters muscle fiber type composition. **a** Immunofluorescence staining using the indicated antibodies or H&E staining of TA treated with AGR-Scr or AGR-*H19*-Rgof. Scale bars, 1 mm or 100 μm as indicated. **b** Number of muscle fibers per muscle piece of animals with indicated treatment. Mean ± SD, *n* = 5 animals per experimental group, Student’s *t* test. **c** Frequency distribution of cross-sectional TA-muscle fiber area. Mean ± SD, *n* = 5 animals per experimental group. **d** SDH staining (top) and immunostaining of different skeletal muscle fiber-types (bottom) from TA of animals with indicated treatment. Scale bars, 1 mm. **e**, **f** Percentage of different skeletal muscle fiber-types from QUAD (**e**) or SOL (**f**) of animals with indicated treatment. Mean ± SD, *n* = 5 animals per experimental group, Student’s *t* test. **g** SDH-positive fibers per muscle piece of animals with indicated treatment. Mean ± SD, *n* = 5 animals per experimental group, Student’s *t* test. **h** Forelimb grip strength test of male (left) or female (right) animals with indicated treatment. Mean ± SD, *n* = 8 animals per experimental group, Student’s *t* test. **i** Running speed of male (left) or female (right) animals with indicated treatment. Mean ± SD, *n* = 15 animals per experimental group, Student’s *t* test. **j** Top: graphic illustration of sprint treadmill protocol. Bottom: Maximum speed of male (left) or female (right) animals with indicated treatment. Mean ± SD, *n* = 10 animals per experimental group, Student’s *t* test

Histological analysis and immunofluorescent staining suggested that AGR-*H19*-Rgof treated soleus (SOL), extensor digitorum longus (EDL), TA, gastrocnemius (GAS), and QUAD showed an increased number of muscle fibers and fiber area compared to Scr mimics-treated TA (Fig. 6a–c and Additional file 4: Fig. S6a). To analyze the fiber composition of slow and fast twitch muscles in AGR-Scr and AGR-*H19*-Rgof-treated mice, we immunolabeled the aforementioned muscles pieces with antibodies recognizing specific myosin-heavy chain isoforms, finding that AGR-*H19*-Rgof-treated QUAD, SOL, and TA exhibit a substantial shift toward the production of fast glycolyctic fiber type IIb and a reduced percentage of fast oxidative fiber type IIa (Fig. 6d, e and Additional file 4: Fig. S6b). SOL showed marginally reduced type I and increased type II fibers (Fig. 6f). The histochemical assay for Succinic Dehydrogenase (SDH) staining was used to distinguish between oxidative and low-oxidative muscle fibers [47], finding that AGR-*H19*-Rgof treated animals exhibited increased SDH+ muscle staining in SOL and EDL muscles; minimally altered SDH+ in TA and GAS; and reduced SDH+ fibers in QUAD (Fig. 6d, g). Our data suggested that the AGR-*H19*-Rgof treatment alters muscle fiber type components, which may affect the performance of the animals.

Type II fibers are correlated with muscle strength. Therefore, we examined the exercise capacity and endurance of AGR-*H19*-Rgof-treated animals. Compared to AGR-Scr, both male and female AGR-*H19*-Rgof-treated animals exhibited increased average force (Fig. 6h). Treadmill exhaustion tests indicated that AGR-*H19*-Rgof-treated animals showed increased speed compared to age and gender-matched Scr-treated controls (Fig. 6i). We further performed sprint interval training using a treadmill (Fig. 6j). AGR-*H19*-Rgof-treated mice exhibited elevated maximum running speed compared to control groups for both male and female animals (Fig. 6j). Taken together, our data suggested that AGR-*H19*-Rgof mimics may lead to muscular hypertrophy, increased type II muscle fiber percentage, and improved speed and strength in these animals.

Furthermore, AGR-*H19*-Rgof-treated mice exhibited minimally affected left ventricular (LV) end diastolic diameter, LV stroke volume, LV ejection fraction, LV fractional shortening, and interventricular septal end diastole compared to the AGR-Scr-treated group (Additional file 4: Fig. S7a-g). Hence, our findings suggested that treatment with AGR-*H19*-Rgof is unlikely to result in heart hypertrophy. We further surveyed the status of the diaphragm and aorta tunica media, finding that the AGR-*H19*-Rgof treated diaphragms exhibited increased thickness compared to AGR-Scr-treated groups (Additional file 4: Fig. S7h-i). The AGR-*H19*-Rgof treatment resulted in unaltered aorta tunica media and tunica adventitia thickness (Additional file 4: Fig. S7h, j, k). Hence, our findings suggested that the AGR-*H19*-Rgof treatment is unlikely to affect the function of cardiac muscle and smooth muscle.

Skeletal muscle is a major organ in energy production and glucose consumption. CLAMS analysis indicated that AGR-*H19*-Rgof-treated animals exhibit elevated respiratory exchange ratio (RER) compared to gender and age-matched AGR-Scr-treated control animals, suggesting that AGR-*H19*-Rgof-treated animals exhibit elevated CO_2_ production and O_2_ consumption (Additional file 4: Fig. S8a-b). The total food intake and activity per day/night of the AGR-*H19*-Rgof-treated mice were not significantly altered compared to the control group (Additional file 4: Fig. S8c-d). Furthermore, AGR-*H19*-Rgof-treated animals exhibited reduced total cholesterol, serum triglyceride concentrations, and blood glucose levels in response to a glucose or insulin challenge (Additional file 4: Fig. S8e-h). When fed with a regular diet (Chow), AGR-*H19*-Rgof-treated animals also showed reduced fatty acid accumulation in the liver and reduced adipocyte size in WAT compared to AGR-Scr-treated animals (Additional file 4: Fig. S8i-k). These data suggested that AGR-*H19*-Rgof-treated animals exhibit potential resistance to obesity in addition to muscular hypertrophy.

### The anti-obesity effect of AGR-*H19*-Rgof lncRNA mimics

**Fig. 7:**
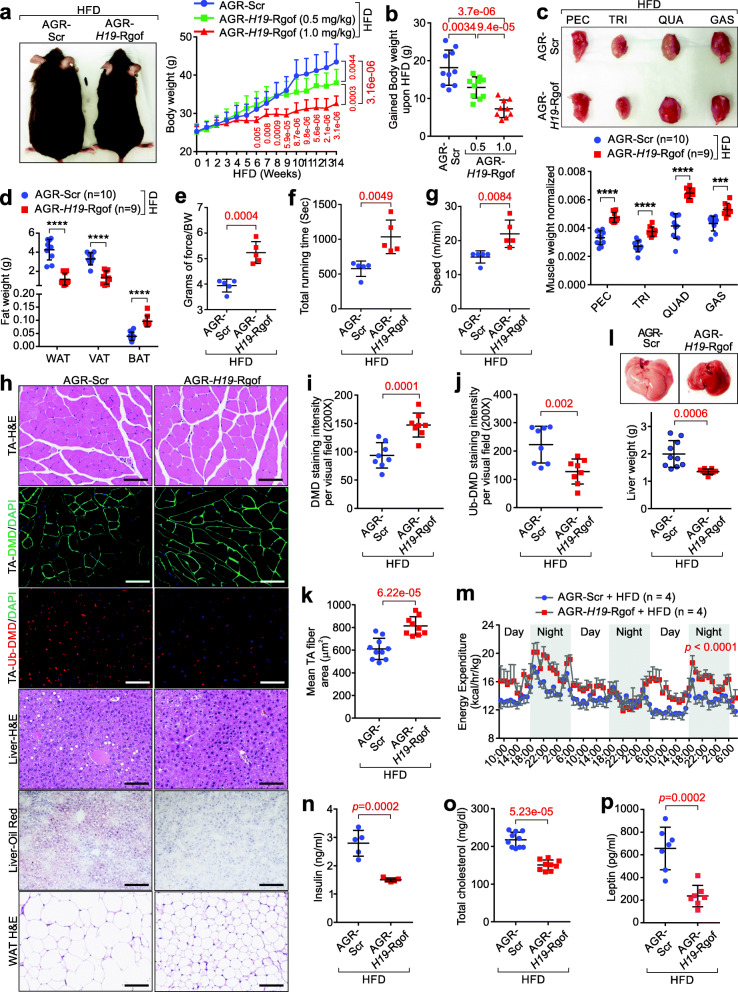
AGR-*H19*-Rgof alleviates HFD-induced obesity. **a** Left: Representative pictures of C57BL/6 J mice on a HFD followed by indicated treatment. Right: Comparison of body weights of mice with indicated treatment. Mean±SD, n=10 animals per group, one-way ANOVA. **b** Gained body weight after 14 weeks on HFD followed by indicated treatment. Mean ± SD, *n* = 10 animals per group, one-way ANOVA. **c** Top: Representative images showing individual muscle pieces. Bottom: Normalized weights of individual muscles. Mean ± SD, *n* = 10, 9 animals per group, Student’s *t* test. **d** Weight measurements of fat tissues. Mean±SD, *n* = 10, 9 animals per group, Student’s *t* test. **e** Forelimb grip strength test of C57BL/6 J mice on a HFD followed by indicated treatment. Mean ± SD, *n* = 5 animals per group, Student’s *t* test. **f**, **g** Total running time (**f**) or speed (**g**) of C57BL/6 J mice on a HFD followed by indicated treatment. Mean±SD, *n* = 5 animals per group, Student’s *t* test. **h** H&E, immunofluorescence staining, and Oil Red O staining of TA, livers, and WAT of C57BL/6 J mice on a HFD followed by indicated treatment. Scale bars, 100 μm. **i**, **j** Statistical analysis of staining intensities of DMD (**i**) or Ub-DMD (**j**). Mean ± SD, *n* = 8 animals per group, Student’s *t* test. **k** Mean TA fiber area of C57BL/6 J mice on a HFD followed by indicated treatment. Mean±SD, *n* = 10, 9 animals per group, Student’s *t* test. **l** Representative images (top) and weight measurement (bottom) of livers. Mean ± SD, *n* = 10, 9 animals per group, Student’s *t* test. **m** Energy expenditure measurements of C57BL/6 J mice on a HFD followed by indicated treatment. Mean±SD, *n* = 4 animals per group, two-way ANOVA. **n**–**p** Serum insulin concentration (**n**), total cholesterol (**o**), or leptin concentration (**p**) of C57BL/6 J mice on a HFD followed by indicated treatment. Mean ± SD, *n* = 5, 5 (**n**), 10, 9 (**o**), or 7, 7 (**p**) animals per group, Student’s *t* test. No significance [n.s.], *p* > 0.05, *, *p* < 0.05, **, *p* < 0.01, ***, *p* < 0.001, ***, *p* < 0.0001

We next challenged WT animals with a HFD to determine the anti-obesity effects of the AGR-*H19*-Rgof mimics. The administration of AGR-*H19*-Rgof lncRNA mimics resulted in significantly reduced body weight when using a 0.5 mg/kg or 1 mg/kg dosage (Fig. 7a, b). AGR-*H19*-Rgof lncRNA mimics-treated animals harbored increased skeletal muscle mass and reduced fat mass (Fig. 7c, d). Body composition indicated that animals subjected to the AGR-*H19*-Rgof lncRNA mimics treatment showed increased lean weight and reduced fat weight and fat percentage (Additional file 4: Fig. S9a-e).

Animals administered with AGR-*H19*-Rgof mimics showed increased force output, as revealed by gripping tests, and elevated total running time and speed (Fig. 7e–g). These findings suggested that the administration of AGR-*H19*-Rgof mimics improved the strength and maximum speed of the animals following the HFD challenge. Furthermore, the histological analysis indicated that animals subjected to AGR-*H19*-Rgof mimics exhibited increased expression of DMD and concurrently reduced Ub-DMD and increased fiber area in TA (Fig. 7h–k). Treatment with AGR-*H19*-Rgof mimics significantly reduced fatty acid deposition in the liver and WAT (Fig. 7h, l).

CLAMS analysis indicated that following AGR-*H19*-Rgof lncRNA mimics treatment, the animals exhibited elevated energy expenditure, although the overall food intake and the total activity of these animals were similar to the animals subjected to the AGR-Scr and HFD challenge (Fig. 7 m and Additional file 4: Fig. S9f-g). Treatment with AGR-*H19*-Rgof lncRNA mimics improved glucose metabolism, alleviated insulin resistance, and reduced serum triglyceride concentrations under HFD challenge (Fig. 7n and Additional file 4: Fig. S9h-k). AGR-*H19*-Rgof-treated animals exhibited reduced total cholesterol, triglycerides, and leptin (Fig. 7o, p and Additional file 4: Fig. S9l), suggesting that treatment with AGR-*H19*-Rgof mimics reversed leptin resistance upon HFD challenge. AGR-Scr or AGR-*H19*-Rgof lncRNA mimics-treated animals showed unaltered IGF1 and IGF2 concentrations and undetectable signs of toxicity following blood chemistry analysis (Additional file 4: Fig. S9m-p).

We further treated the male *Lep*^ob/ob^ mice with AGR-Scr or AGR-*H19*-Rgof mimics, finding that the animals treated with AGR-*H19*-Rgof exhibited reduced body weight 8 weeks post-treatment (Additional file 4: Fig. S10a). *Lep*^ob/ob^ mice subjected to AGR-*H19*-Rgof mimics harbored increased muscle mass compared to the AGR-Scr group (Additional file 4: Fig. S10b). The administration of AGR-*H19*-Rgof mimics significantly improved fatty acid deposition in *Lep*^ob/ob^ livers (Additional file 4: Fig. S10c-d).

Taken together, our findings suggested that *H19*-GOF plays important roles in modulating the metabolic balance of skeletal muscles, leading to muscular hypotrophy and resistance to HFD or *leptin* resistance-induced obesity.

## Discussion

Non-hormonal-based performance enhancers remain largely elusive. An efficient yet safe strategy for enhancing muscle building that also combats obesity may serve as a promising therapeutic option for obese patients. Particularly, for sarcopenic obesity, overcoming muscle wasting may significantly prevent or improve the survival rate of obesity-related complications in these patients [48]. We identified that *H19* directly and specifically associated with DMD’s ZnF domain. The GOF mutant of *H19* exhibited enhanced binding affinity to DMD protein, leading to DMD stabilization. Inhibition of MRCKα and SNCA-mediated DMD phosphorylation facilitated the interaction between *H19* and DMD and further stabilized DMD protein. Hence, our findings demonstrated the molecular mechanisms of underlying the phosphorylation of DMD.

DMD plays vital roles in skeletal muscle differentiation and regeneration [49, 50]. DMD has also been suggested to play important roles in modulating the metabolism of skeletal muscle, with enlarged skeletal muscle mass modulating adipose metabolism in turn [51]. Genetic evidence indicated that mdx mice exhibit alterations in fatty acid metabolism and reprogrammed glucose uptake [52]. DMD deficiency leads to an aberrant mitochondrial and metabolic phenotype prior to the onset of myofiber necrosis [53, 54]. Furthermore, the DMD glycoprotein complex is physically and functionally linked to the insulin receptor on the plasma membrane [55]. Impaired cytoskeletal anchoring of DMD may lead to insulin resistance [56]. Mutations or deletions of the *DMD* gene lead to muscular dystrophy, which currently lacks viable therapeutic treatment strategies [57–59]. Hence, stabilization of DMD might enhance skeletal muscle differentiation and improve glucose metabolism. Whether AGR-*H19*-Rgof could serve as an alternative therapeutic strategy for muscle dystrophy in DMD patients is under further investigation. The potential off-target effects of AGR-*H19*-Rgof were largely ruled out using unlabeled *H19*-Rgof. However, the physiological impact of AGR-*H19*-Rgof requires further evaluation before it can be translated to clinically-orientated applications.

*H19* is a long noncoding RNA that is regulated by the imprinted gene network [60, 61]. Manipulating the expression levels of *H19* co-regulates the expression status of IGF2 [62, 63]. Hence, elevated IGF2 contributes to the muscular hypertrophy phenotype in *H19* knockout or truncation-harboring mice. In order to clarify the functional role of *H19* in skeletal muscle without the effects caused by altered IGF2 status, knock-in of a single or a small number of LOF or GOF mutations is necessary to clarify the *bona fide* biological effects of *H19*. Using *in vitro* myoblast differentiation to myotubes, our findings suggested that the presence of the *H19*-GOF mutant facilitates myoblast differentiation, which is consistent with the previous observation that overexpression of *H19* facilitates muscle regeneration and that knock-out of *H19* impairs muscle differentiation [64, 65]. Utrophin shares structural and functional similarities with DMD. It is possible that AGR-*H19*-Rgof exhibits moderate interactions with the ZnF domain of utrophin. The expression of utrophin exhibits low tissue specificity [66]. Utrophin has been previously shown to regulate adipocyte droplet size [67]. Hence, the effect of stabilized utrophin on adipose tissues and insulin resistance could be an interesting research direction for further investigation.

The increased type II muscle mass following treatment with AGR-*H19*-Rgof in mice mimics the effect of resistance exercise training, which also demonstrates type II fiber hypertrophy [68]. Type II muscle fiber loss/atrophy is also an important contributing factor in the development of muscle weakness during aging [69, 70], which suggests that patients with sarcopenia could also benefit from an *H19*-Rgof treatment. A number of studies have shown that slow twitch/Type I muscle express more DMD than fast twitch/Type II muscle [71], and fast twitch muscle fibers are preferentially affected in DMD patients [72]. These findings suggest that Type II muscles are more prone to being affected by the level of DMD expression and the stability of DMD complexes. There is also evidence showing that TRIM63 is preferentially expressed in type II fibers [73]. We previously reported that the stabilization of DMD complexes by *H19*-GOF is mediated by TRIM63 [18]. The preferential expression of TRIM63 in type II fibers suggests that AGR-*H19*-Rgof mainly functions in type II fibers and results in a selective increase in the number of type II fibers.

Although type I muscle fibers play a pivotal role in glucose/lipid metabolism, there are studies suggesting that increasing the number of type II fibers may also promote favorable effects on glucose metabolism [74]. This is further supported by our observations that treatment with AGR-*H19*-Rgof mitigated the diet-induced obesity. Resistance training, which is associated with an increase in type II fibers, has also been recommended as a complementary exercise modality for insulin-resistant patients. There are also studies showing that increased glycolytic muscle mass in obese mice leads to a reduction in WAT and improved metabolic parameters [75]. Thus, a modest increase in glycolytic muscle can also have a profound effect on whole body metabolism and adipose mass, which is consistent with our findings.

## Conclusions

We report that *H19* GOF exhibited enhanced association with DMD protein, which was subjected to MRCKα and SNCA-mediated phosphorylation. *H19*-GOF mutants promoted the differentiation and fusion of myotubes. The AGR-*H19*-Rgof RNA mimics enhanced the physical performance of our mice and antagonized HFD- and *leptin* deficiency-induced obesity.

## Supplementary Information

**Additional file 1.** Table S1: Oligonucleotides used in this study.

**Additional file 2.** Table S2: Clinical information of tissue samples and hiPSCs used in this study.

**Additional file 3.** Table S3: List of antibodies used in this study.

**Additional file 4. **Supplementary Figures 1-10 and figure legends. Fig. S1. Characterization of *H19* expression in human tissues. Fig. S2. MRCKα and SNCA inhibitors attenuate the poly-ubiquitination of DMD in BMD. Fig. S3. *H19*-GOF facilitates myotube differentiation. Fig. S4. *H19*-Rgof associates with the utrophin zinc finger domain. Fig. S5. AGR-*H19*-Rgof improves animal performance. Fig. S6. AGR-*H19*-Rgof treatment alters the muscle fiber types. Fig. S7. Minimal effect of AGR-*H19*-Rgof in mouse cardiac muscle and tunica media. Fig. S8. AGR-*H19*-Rgof enhances aerobic metabolism. Fig. S9. AGR-*H19*-Rgof attenuates HFD-induced obesity. Fig. S10. AGR-*H19*-Rgof inhibits *leptin* deficiency-induced obesity.

**Additional file 5. **Table S4: Identification of *H19*-binding proteins by mass spectrometry.

**Additional file 6.** Table S5: Characterization of DMD post-translational modifications.

**Additional file 7. **Table S6: Laboratory parameters of animals subjected to AGR-Scr or AGR-*H19*-Rgof treatment.

## Data Availability

All data generated or analyzed during this study are included in this published article and its supplemental information files.

## Abbreviations

lncRNAs: Long noncoding RNAs
AGR-*H19*-Rgof: Agrin tagged *H19* RNA gain-of-function
CNS: Central nervous system
polyUb: Poly-ubiquitination
GOF: Gain-of-function
LOF: Loss-of-function
Rgof: RNA gain-of-function
Rlof: RNA loss-of-function
SMCC: Succinimidyl 4-(N-maleimidomethyl)cyclohexane-1-carboxylate
iPSC: Induced pluripotent stem cells
HSMC: Human smooth muscle cells
ITT: Insulin tolerance test
GTT: Glucose tolerance test
TC: Total cholesterol
TG: Triglycerides
ALT: Alanine aminotransferase
AST: Aspartate aminotransferase
H&E: Hematoxylin and eosin
BAT: Brown adipose tissue
WAT: White adipose tissue
VAT: Visceral adipose tissue
SDH: Succinic dehydrogenase
EMSA: Electrophoretic mobility shift assay
CLIP: Crosslinking and immunoprecipitation
RIP: RNA immunoprecipitation
KO: Knock out
SDS-PAGE: Sodium dodecyl sulfate polyacrylamide gel electrophoresis
DMD: Dystrophin
NOS1: Nitric oxide synthase
SNTA1: Syntrophin alpha 1
DAG1: Beta-dystroglycan
SGCA: Alpha-sarcoglycan
SNTB1/2: Beta-1-syntrophin/ Beta-2-syntrophin
Scr: Scramble
AChR: Acetylcholine receptor
CBC: Complete blood cell count
SOL: Soleus
EDL: Extensor digitorum longus
TA: Tibialis anterior
GAS: Gastrocnemius
QUAD: Quadriceps
CLAMS: Comprehensive Lab Animal Monitor System
RER: Respiratory exchange ratio
AUC: Areas under the curve
HOMA-IR: Homeostatic Model Assessment of Insulin Resistance
BMD: Becker muscular dystrophy
MRCKα: MRCK alpha
SNCA: α-synuclein
LC-MS: Liquid chromatography–mass spectrometry
BRK: Breast tumor kinase
SC-D: SynuClean-D
BDP: BDP5290
ZnF: Zinc finger
