# Supplementary material for: Functional significance of gain-of-function H19 lncRNA in skeletal muscle differentiation and anti-obesity effects
Source: Genome Med. 2021 Aug 28;13:137. doi: 10.1186/s13073-021-00937-4 (PMC8403366; doi:10.1186/s13073-021-00937-4)
Supplement: Supplementary file 4 — Additional file 4. Supplementary Figures 1-10 and figure legends. Fig. S1. Characterization of H19 expression in human tissues. Fig. S2. MRCKα and SNCA inhibitors attenuate the poly-ubiquitination of DMD in BMD. Fig. S3. H19-GOF facilitates myotube differentiation. Fig. S4. H19-Rgof associates with the utrophin zinc finger domain. Fig. S5. AGR-H19-Rgof improves animal performance. Fig. S6. AGR-H19-Rgof treatment alters the muscle fiber types. Fig. S7. Minimal effect of AGR-H19-Rgof in mouse cardiac muscle and tunica media. Fig. S8. AGR-H19-Rgof enhances aerobic metabolism. Fig. S9. AGR-H19-Rgof attenuates HFD-induced obesity. Fig. S10. AGR-H19-Rgof inhibits leptin deficiency-induced obesity. [file 13073_2021_937_MOESM4_ESM.docx]

**
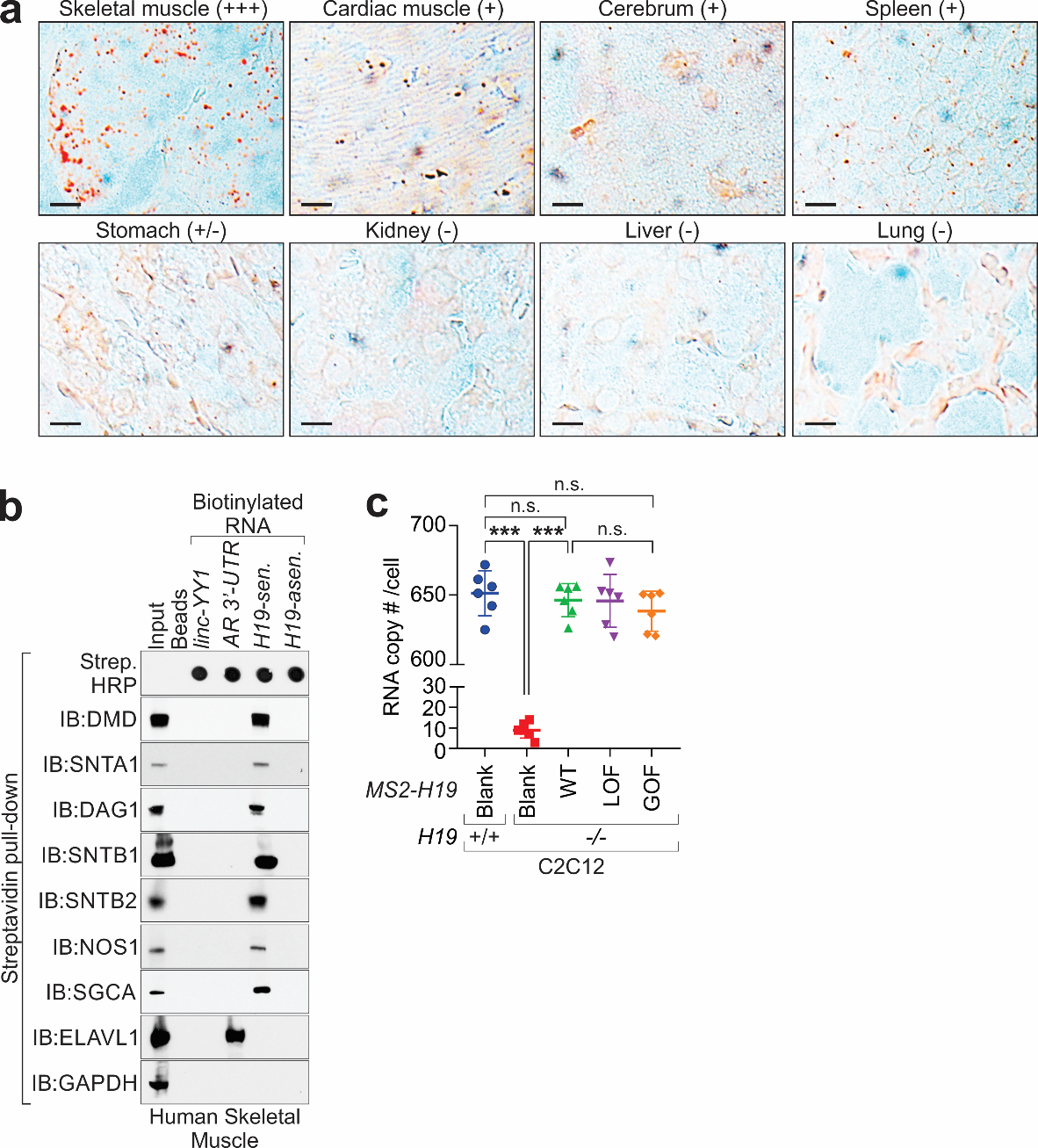
**

**Fig. S1: Characterization of *H19* expression in human tissues.**

(**a**) RNAScope^®^ detection of *H19* expression in human tissue sections including skeleton muscle, heart smooth muscle, cerebrum, spleen, stomach, kidney, liver and lung. Scale bars, 100 µm. (**b**) RNA pulldown using biotinylated RNA as indicated and human skeletal muscle tissues, followed with IB detection using indicated antibodies. (**c**) Human *H19* copy number per cell determined in *H19*-proficient or -deficient C2C12 cells expressing indicated plasmid. Mean±SD, n=3 independent experiments, one-way ANOVA. No significance [n.s.], *p* > 0.05, *, *p* < 0.05, **, *p* < 0.01, ***, *p* < 0.001.


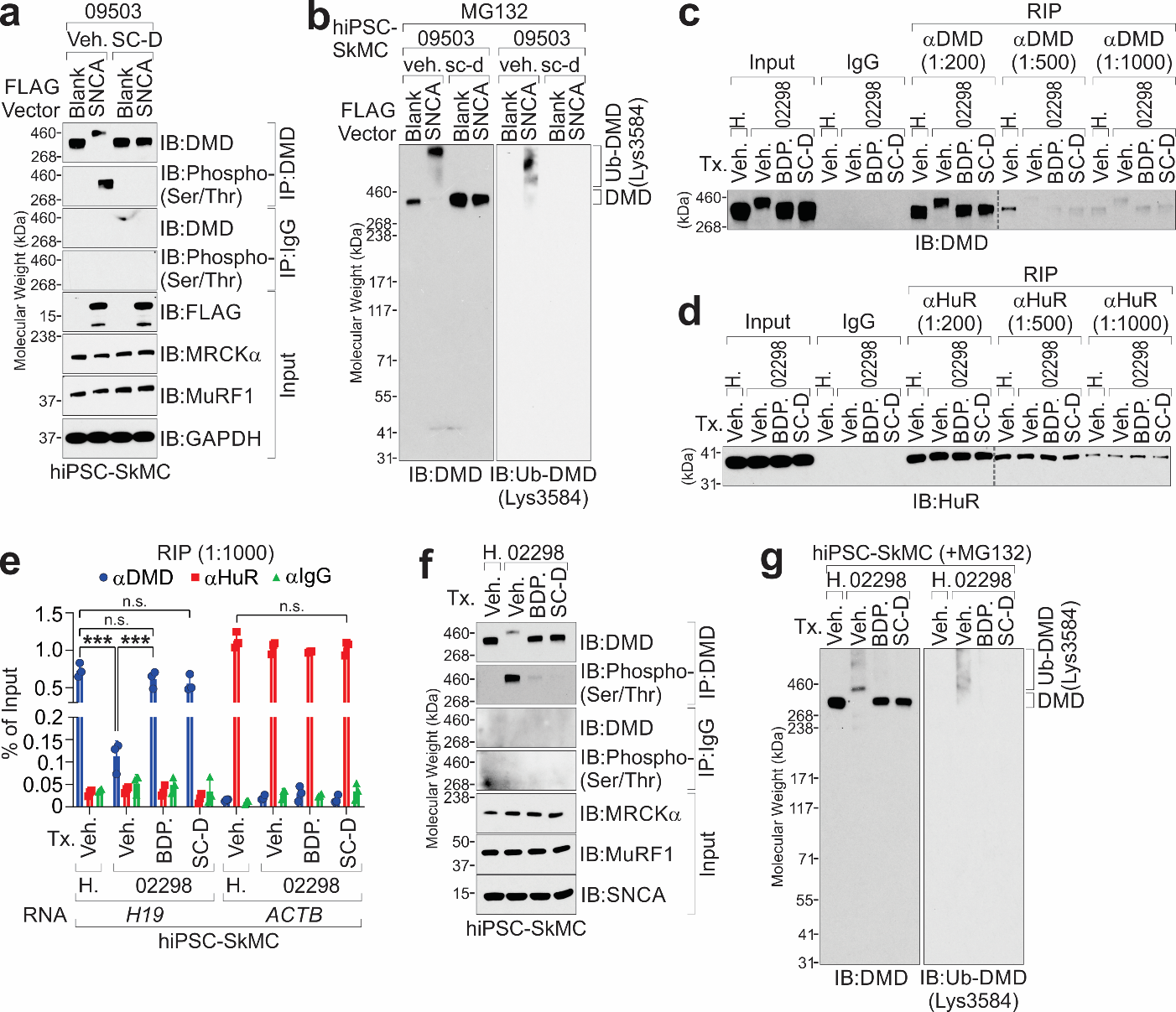


**Fig. S2**. **MRCKα and SNCA inhibitors attenuate the poly-ubiquitination of dystrophin in BMD.** (**a-b)** IB detection using the indicated antibodies in iPSC-SkMC derived from a healthy donor 09503 with exogenous expression of SNCA, followed by vehicle (Veh.) or SynuClean-D (SC-D) treatment (Tx.). (**c-d)** IB detection using the indicated antibodies for input, IgG, or RIP. (**e**) RIP assay using the indicated antibodies of iPSC-SkMC derived from a healthy donor 09503 or BMD patient 02298. Mean±SEM, n=3 independent experiments, one-way ANOVA. (**f-g**) IB detection using the indicated antibodies in iPSC-SkMC derived from a healthy donor 09503 or BMD donors in the presence of the indicated treatment. No significance [n.s.], *p* > 0.05, *, *p* < 0.05, **, *p* < 0.01, ***, *p* < 0.001.


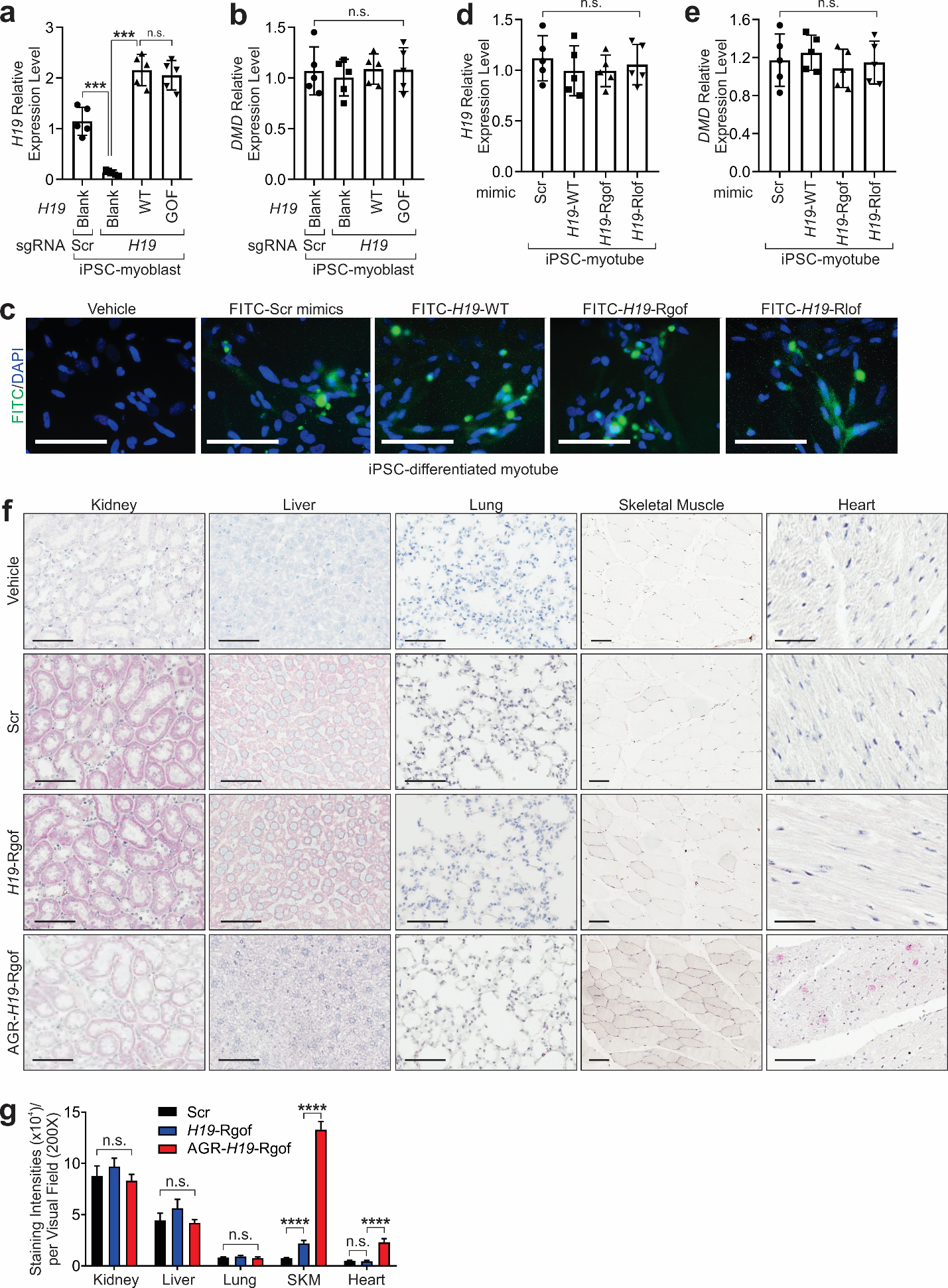


**Fig. S3: *H19*-GOF facilitates myotube differentiation.**

(**a-b**) Relative expression level of *H19* or *DMD* in iPSC-differentiated myotubes expressing *H19* WT or GOF mutant. Mean±SD, n=5 independent experiments, one-way ANOVA (**c**) Immunofluorescence detection of FITC-conjugated RNA mimics as indicated in iPSC-differentiated myotubes. Scale bars, 100 µm. (**d-e**) Relative expression level of *H19* (d) or *DMD* (e) in iPSC-differentiated myotubes delivered with indicated RNA mimics. Mean±SD, n=5 independent experiments, one-way ANOVA. (**f-g**) Representative images (f) or statistical analysis of staining intensities (g) of immunohistological detection of Biotin-labeled AGR-Scr, *H19*-Rgof or AGR-*H19­*-Rgof in kidney, liver, lung, skeletal muscle (quadriceps), and heart 24 hours post administration. Scale bars, 100 μm. Mean±SD, n=5 animals per group, one-way ANOVA. No significance [n.s.], *p* > 0.05, *, *p* < 0.05, **, *p* < 0.01, ***, *p* < 0.001.

**
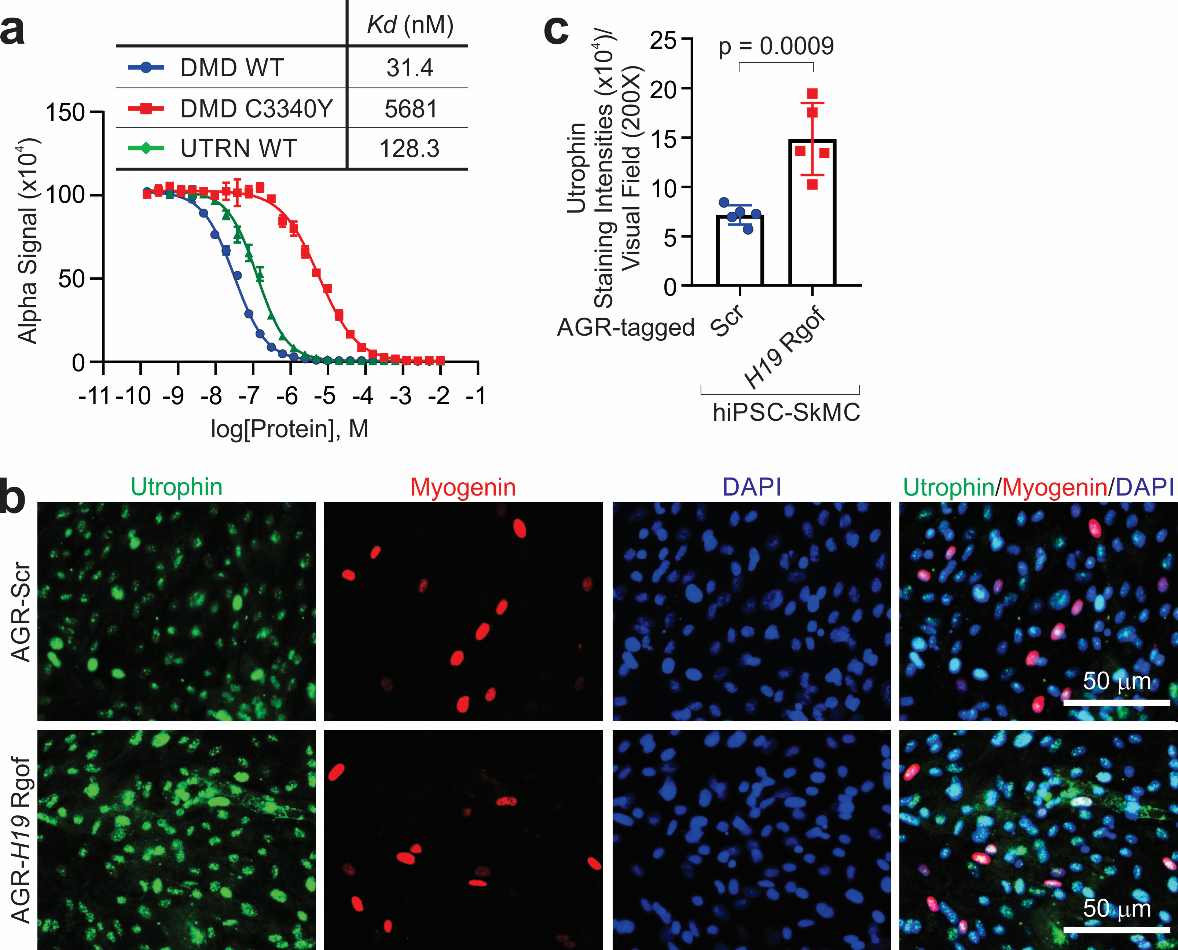
**

**Fig. S4. *H19*-Rgof associates with the utrophin zinc finger domain. (a)** Alpha assay determination of the K_d_ value between *H19*-Rgof and the DMD zinc finger domain (WT), C3340Y mutant, or utrophin zinc finger domain, with the presence of unlabeled DMD WT, C3340Y mutant or utrophin zinc finger domain serving as a competitor. Mean±SD, n=3 independent experiments. (**b-c**) Representative images (b) or statistical analysis of utrophin staining intensities (c) of iPSC-SkMC harboring AGR-Scr or AGR-*H19*-Rgof oligonucleotides. Scale bars, 50 µm. Mean±SD, n=5 independent experiments, Student’s *t* test.

**
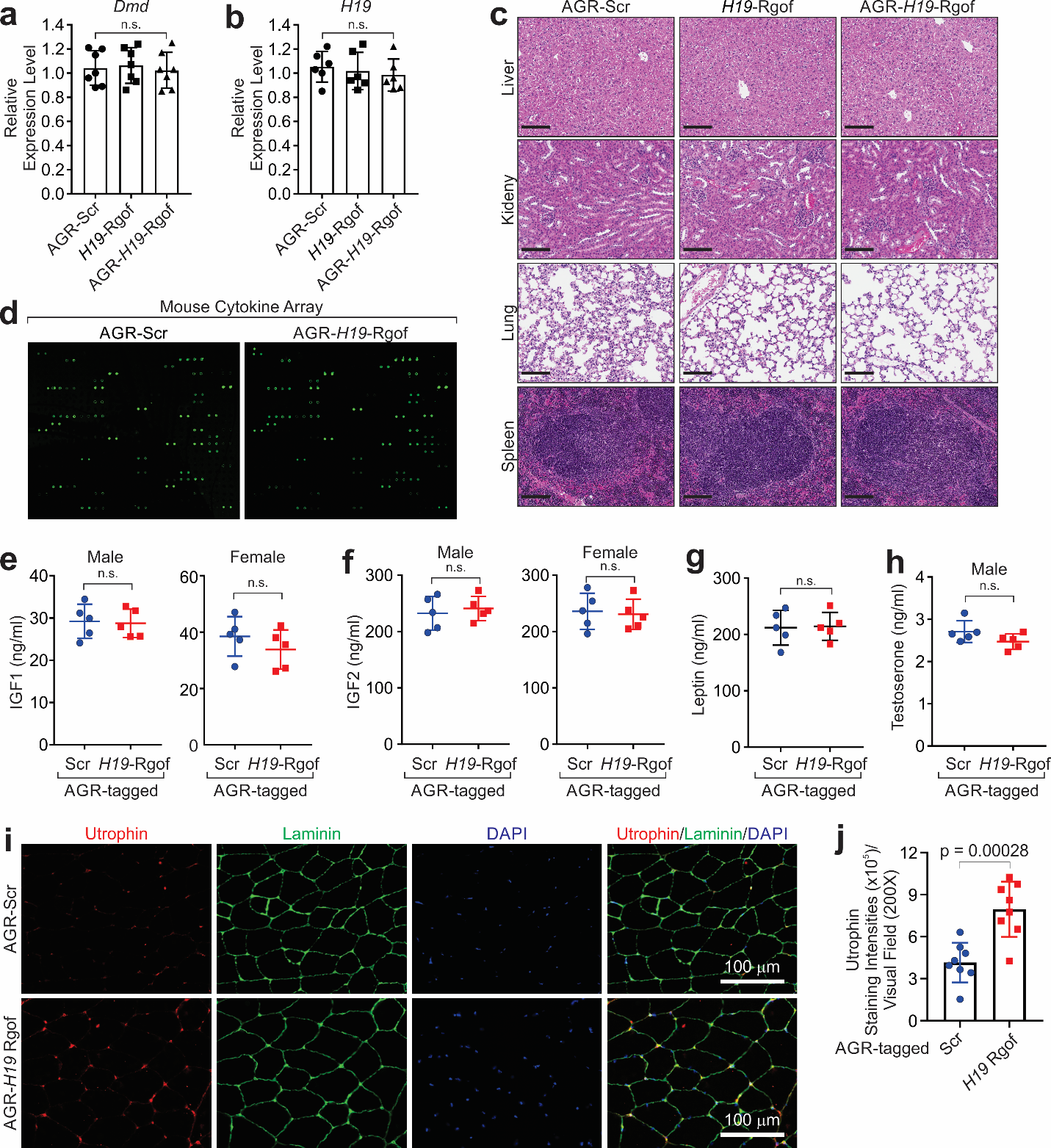
**

**Fig. S5: AGR-*H19*-Rgof improves animal performance.**

**(a**-**b)** Relative expression level of *Dmd* (a) or *H19* (b) in TA of male mice treated with AGR-Scr, *H19*-Rgof or AGR-*H19*-Rgof as indicated. Mean±SD, n=5 independent experiments, one-way ANOVA. (**c**) H&E staining of liver, kidney, lung and spleen of mice indicated treatment. Scale bars, 100 µm. (**d**) Representative images of mouse serum cytokine array of mice with indicated treatment. (**e-f**) Serum IGF1 (e) or IGF2 (f) concentration of male (left) or female (right) mice with indicated treatment. Mean±SD, n=5 independent experiments, Student’s *t* test. (**g-h**) Serum leptin (g) or testosterone (h) concentration of mice with indicated treatment. Mean±SD, n=5 independent experiments, Student’s *t* test. (**i-j**) Representative images (i) or statistical analysis of utrophin staining intensities (j) of animals subjected indicated treatment. Scale bars, 100 µm. Mean±SD, n=8 animals per experimental group, Student’s *t* test. No significance [n.s.], *p* > 0.05, *, *p* < 0.05, **, *p* < 0.01, ***, *p* < 0.001.

**
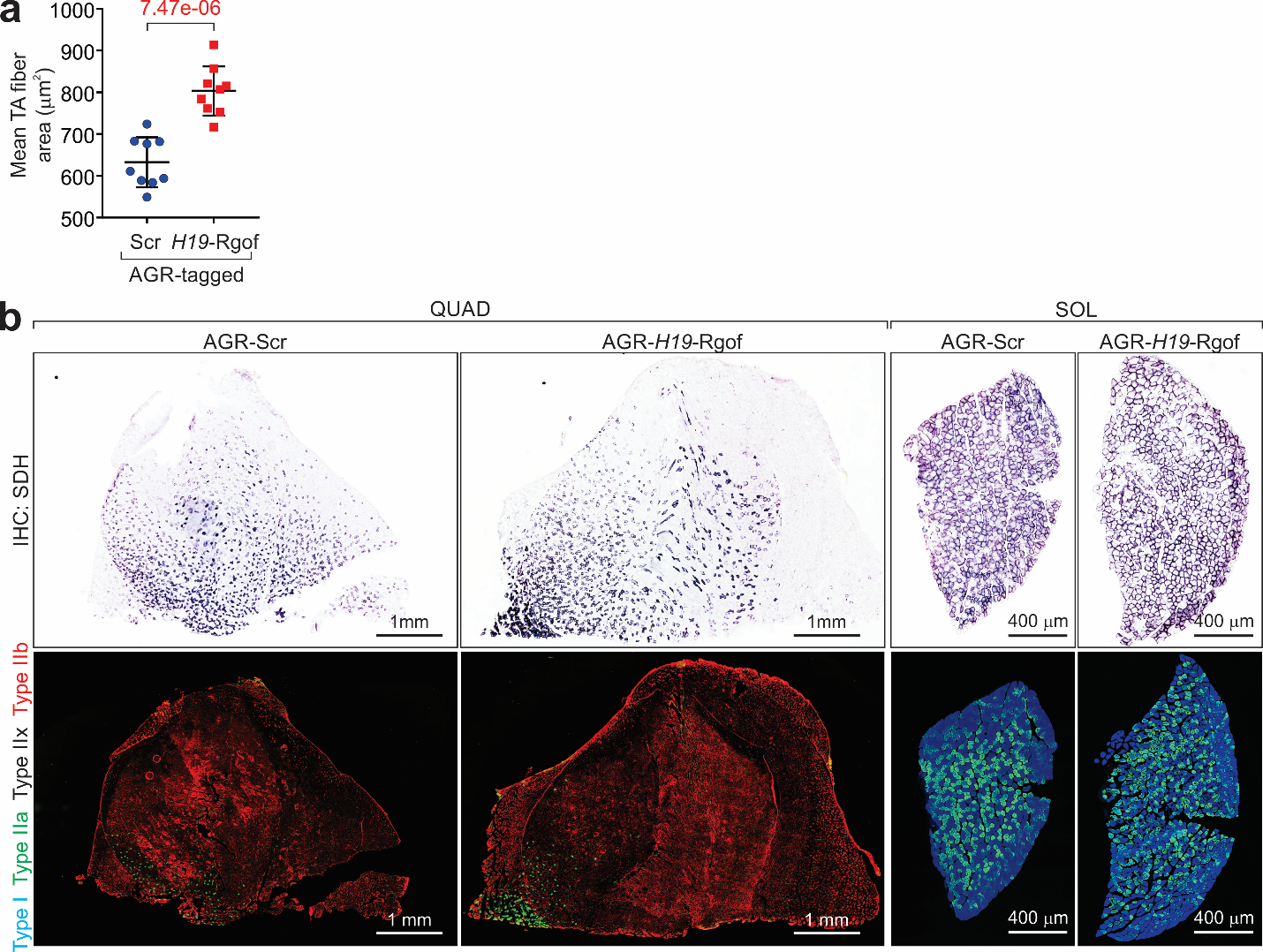
**

**Fig. S6: AGR-*H19*-Rgof treatment alters the muscle fiber types.**

**(a)** Statistical analysis of cross-sectional TA-muscle fiber area. Mean±SD, n=9, 9 animals per experimental group, Student’s *t* test. (**b**) SDH staining (top) and immunolabeling of indicated skeletal muscle fiber-types (bottom) from QUAD and SOL of animals treated with AGR-Scr or AGR-*H19*-Rgof. Scale bars, 1 mm or 400 µm as indicated.

**
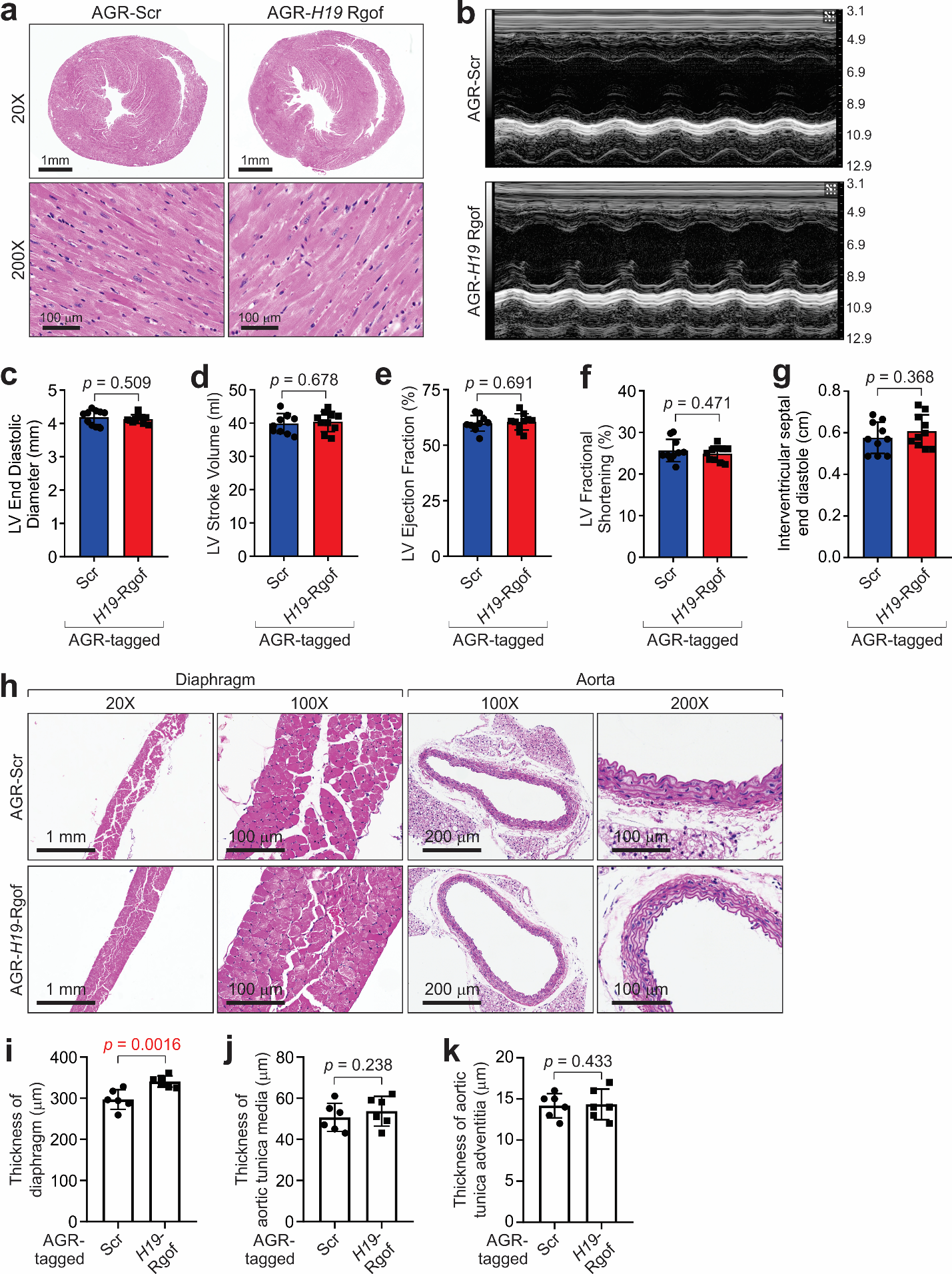
**

**Fig. S7. Minimal effect of AGR-*H19*-Rgof in mouse cardiac muscle and tunica media.**

(**a**) Heart H&E staining from male mice treated with AGR-Scr or AGR-*H19*-Rgof. Scale bars, 1 mm or 100 µm as indicated. (**b-g**) Representative echocardiographic images (b) and echocardiographic parameters of left ventricular (LV) end diastolic diameter (c), LV stroke volume (d), LV ejection fraction (e), LV fractional shortening (f), and interventricular septal end diastole (g) of mice with indicated treatment. Mean±SD (c-g), n=10, 10 animals, Student’s *t* test. (**h-k**) Representative histological images (h) and thickness measurement of indicated parameters (i-k) of mice with indicated treatment. Scale bars, 1 mm, 100 µm or 200 µm. Mean±SD (i-k), n=6, 6 animals, Student’s *t* test.

**
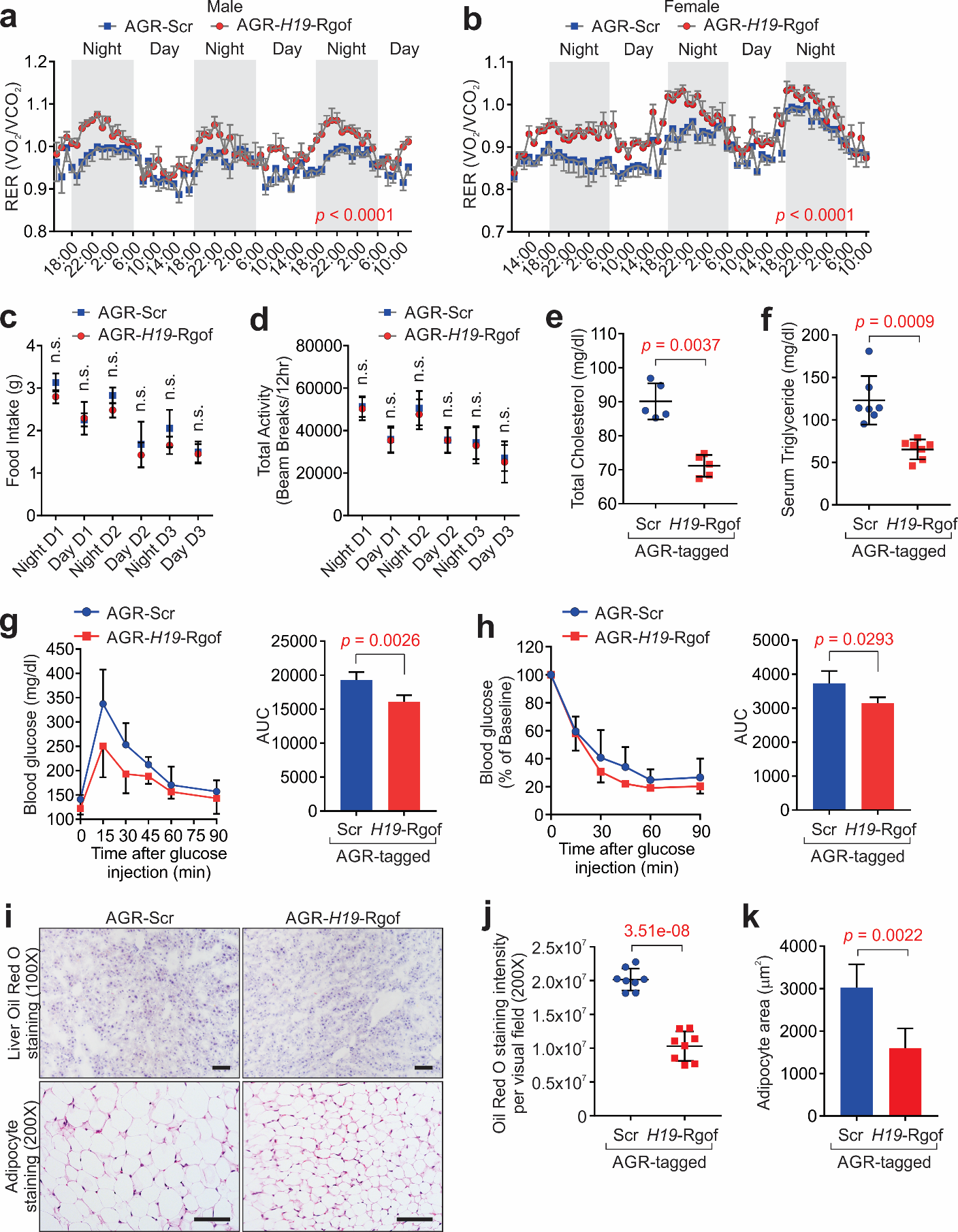
**

**Fig. S8: AGR-*H19*-Rgof enhances aerobic metabolism.**

(**a** and **b**) CLAMS measurement of RER in male (a) or female (b) mice treated with indicated treatment. Mean±SD, n=4 animals per group, two-way ANOVA. (**c**) Comparison of food consumption (grams consumed per 12 h) of male mice with indicated treatment. Mean±SD, n=4 animals per group, Student’s *t* test. (**d**) Total activity during both the light and dark cycles determined by CLAMS of male mice treated with indicated treatment. Mean ± SEM, n = 4 animals per group, Student’s *t* test. (**e**-**f**) Total serum cholesterol concentration (e) or triglyceride concentration (f) of mice with indicated treatment. Mean±SD, n=5 (e) or 7 (f) animals per group, Student’s *t* test. (**g**) Left: GTT was performed on male mice with indicated treatment. Mean±SD, n=5 animals per group. Right: area under the curve (AUC) determination. Mean±SD, n=5 animals per group, Student’s *t* test. (**h**) Left: ITT was performed on male mice with indicated treatment. Mean±SD, n=5 animals per group. Right: area under the curve (AUC) determination. Mean±SD, n=5 animals per group, Student’s *t* test. (**i**) Oil Red O staining staining of liver (top) and H&E staining of WAT (bottom) of male mice with indicated treatment. Scale bars, 100 µm. (**j**) Statistical analysis of Oil Red O staining of male mice with indicated treatment. Mean±SD, n=8 animals per group, Student’s *t* test. (**k**) Statistical measurement of adipocyte area of WAT of male mice with indicated treatment. Mean±SD, n=5 animals per group, Student’s *t* test. No significance [n.s.], *p* > 0.05, *, *p* < 0.05, **, *p* < 0.01, ***, *p* < 0.001.

**
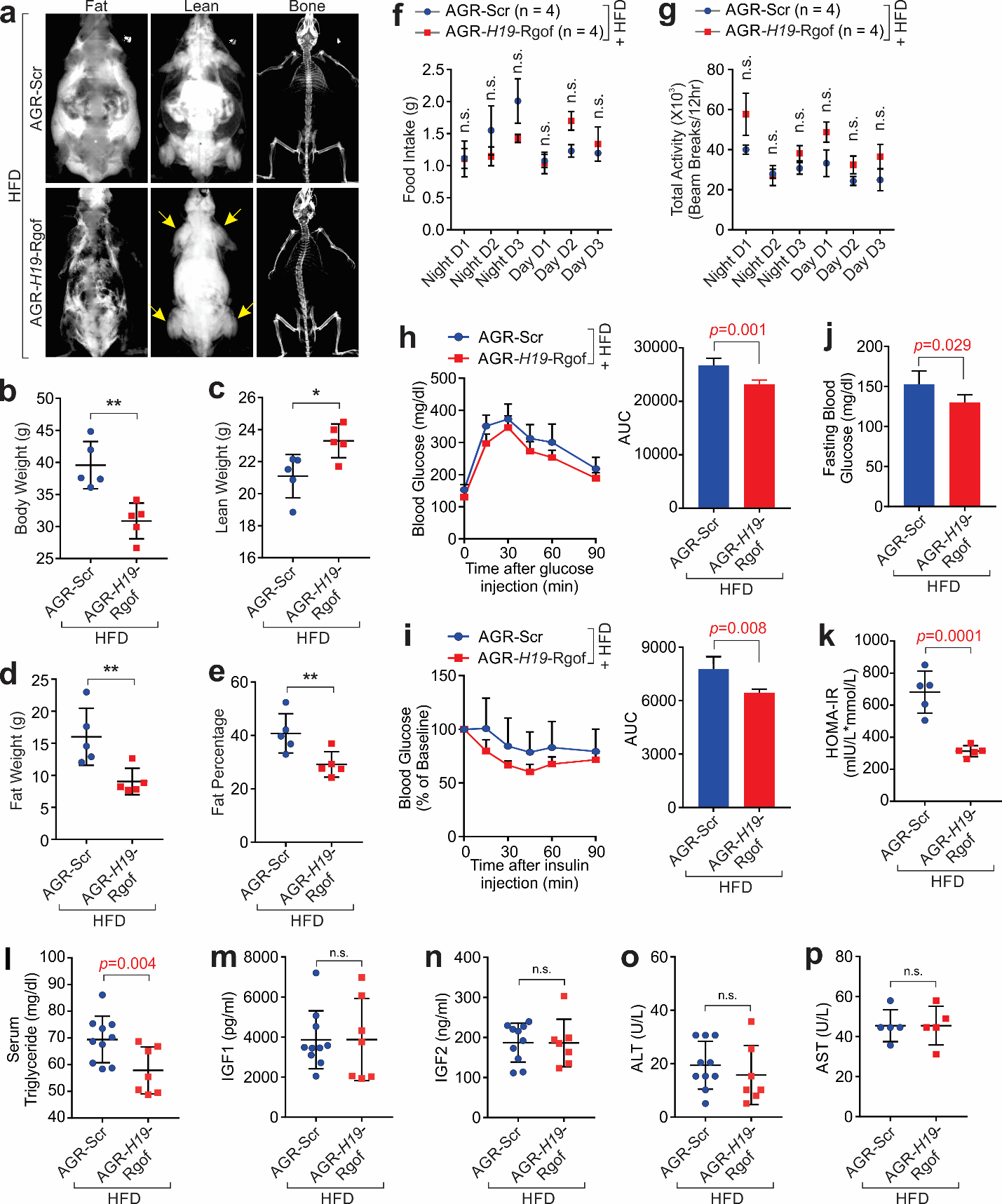
**

**Fig. S9: AGR-*H19*-Rgof attenuates HFD-induced obesity.**

(**a**) Representative pictures of lean, fat and bone tissues by the dual energy x-ray absorptiometry imaging system from male mice on HFD followed with AGR-Scr or AGR-*H19*-Rgof treatment. (**b-e**) Quantification of body weight (b), lean weight (c), fat weight (d), and fat percentage (e) by the dual energy x-ray absorptiometry imaging system from mice with indicated treatment. Mean±SD, n=5 animals per experimental group, Student’s *t* test. (**f**) Comparison of food consumption (grams consumed per 12 h) of male mice on HFD with indicated treatment. Mean±SD, n=4 animals per group, Student’s *t* test. (**g**) Total activity during both the light and dark cycles determined by CLAMS. Mean±SD, n = 4 animals per group, Student’s *t* test. (**h**) Left: GTT was performed on male mice on HFD with indicated treatment. Mean±SD, n=5 animals per group. Right: area under the curve (AUC) determination. Mean±SD, n=5 animals per group, Student’s *t* test. (**i**) Left: ITT was performed on male mice on HFD with indicated treatment. Mean±SD, n=5 animals per group. Right: area under the curve (AUC) determination. Mean±SD, n=5 animals per group, Student’s *t* test. (**j**) Serum glucose concentration under fasting condition of male mice on HFD with indicated treatment. Mean±SD, n=5 animals per group, Student’s *t* test. (**k-p**) HOMA-IR (k), serum triglyceride (l), IGF1 (m), IGF2 (n), ALT (o) or AST (p) of male mice on HFD with indicated treatment. Mean±SD, n=5, 5 (k), 10, 7 (l-o), 5, 5 (p) animals per group, Student’s *t* test. No significance [n.s.], *p* > 0.05, *, *p* < 0.05, **, *p* < 0.01, ***, *p* < 0.001.


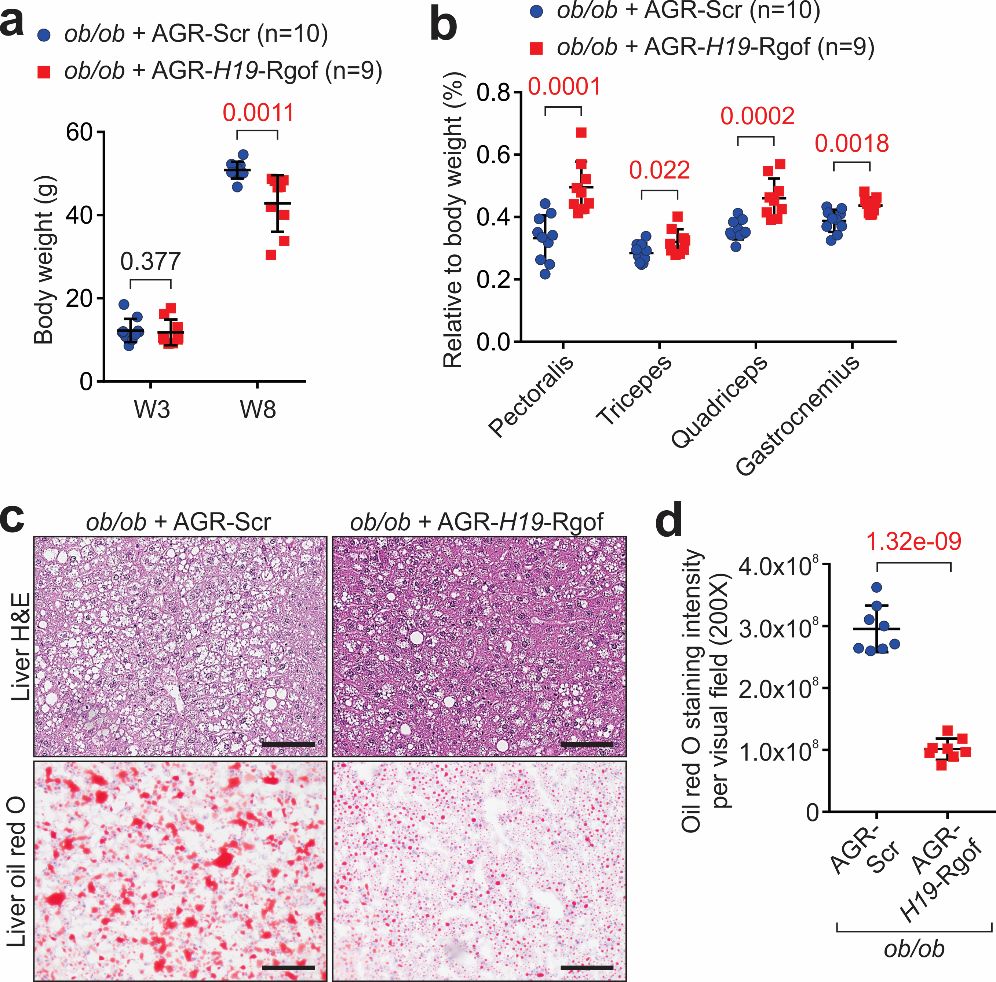


**Fig. S10: AGR-*H19*-Rgof inhibits *leptin* deficiency-induced obesity.**

(**a**) Body weight of male *Lep^Ob/Ob^* mice before (3-week old) and after AGR-Scr or AGR-*H19*-Rgof treatment (8-week old). Mean±SD, n=10, 9 animals per group, Student’s *t* test. (**b**) Normalized weights of individual muscles from *Lep^Ob/Ob^* mice treated with AGR-Scr or AGR-*H19*-Rgof for 5 weeks. Mean±SD, n=10, 9 animals per group, Student’s *t* test. (**c**) Representative images of H&E staining and Oil Red O staining of liver sections from *Lep^Ob/Ob^* mice with indicated treatment. Scale bars, 100 µm. (**d**) Statistical analysis of Oil Red O staining intensity of liver sections from *Lep^Ob/Ob^* mice with indicated treatment. Mean±SD, n=8, 8 animals per group, Student’s *t* test.
